# The global, regional, and national burden and quality of care index (QCI) of colorectal cancer; a global burden of disease systematic analysis 1990–2019

**DOI:** 10.1371/journal.pone.0263403

**Published:** 2022-04-21

**Authors:** Seyed Aria Nejadghaderi, Shahin Roshani, Esmaeil Mohammadi, Moein Yoosefi, Negar Rezaei, Zahra Esfahani, Sina Azadnajafabad, Naser Ahmadi, Sarvenaz Shahin, Ameneh Kazemi, Alireza Namazi Shabestari, Ardeshir Khosravi, Ali H. Mokdad, Bagher Larijani, Farshad Farzadfar

**Affiliations:** 1 Non-Communicable Diseases Research Center, Endocrinology and Metabolism Population Sciences Institute, Tehran University of Medical Sciences, Tehran, Iran; 2 Sina Trauma and Surgery Research Center, Tehran University of Medical Sciences (TUMS), Tehran, Iran; 3 Endocrinology and Metabolism Research Center, Endocrinology and Metabolism Clinical Sciences Institute, Tehran University of Medical Sciences, Tehran, Iran; 4 Department of Biostatistics, University of Social Welfare and Rehabilitation Sciences, Tehran, Iran; 5 Department of Geriatric Medicine, School of Medicine, Tehran University of Medical Sciences, Tehran, Iran; 6 Iranian Ministry of Health and Medical Education, Tehran, Iran; 7 Institute for Health Metrics and Evaluation, University of Washington, Seattle, WA, United States of America; University of Central Florida, UNITED STATES

## Abstract

**Background:**

Colorectal cancer (CRC) is among the five most incident and lethal cancers in world and its burden varies between countries and sexes. We aimed to present a comprehensive measure called the quality of care index (QCI) to evaluate the inequity and healthcare quality of care regarding CRC by sex and location.

**Methods:**

Data on the burden of CRC were extracted from the Global Burden of Disease study 2019. It was transformed to four ratios, including mortality-to-incidence, disability-adjusted life years (DALYs)-to-prevalence, prevalence-to-incidence, and years of life lost (YLLs)-to-years lived with disability (YLDs). Principal component analysis was implemented on the four ratios and the most influential component was considered as QCI with a score ranging from zero to 100, for which higher scores represented better quality of care. Gender Disparity Ratio (GDR) was calculated by dividing QCI for females by males.

**Results:**

The global incidence and death numbers of CRC were 2,166,168 (95% uncertainty interval: 1,996,298–2,342,842) and 1,085,797 (1,002,795–1,149,679) in 2019, respectively. Globally, QCI and GDR values were 77.6 and 1.0 respectively in 2019. There was a positive association between the level of quality of care and socio-demographic index (SDI) quintiles. Region of the Americas and African Region had the highest and lowest QCI values, respectively (84.4 vs. 23.6). The QCI values started decreasing beyond the age of 75 in 2019 worldwide.

**Conclusion:**

There is heterogeneity in QCI between SDI quintiles. More attention should be paid to people aged more than 75 years old because of the lower quality of care in this group.

## Introduction

Colorectal cancer (CRC) was the second cause of death and the third one in incidence among cancers which constituted 9.4% and 10.0% of deaths and incidence of all cancers, respectively, among both sexes and around the world in 2020 [1]. Disability-adjusted life years (DALYs) for CRC out of total DALYs attributable to all causes has increased from 0.48% in 1990 to 0.96% in 2019 [2].

The quality of care according to the World Health Organization (WHO) definition is “the extent of which healthcare services are provided to individuals and populations to improve desired health outcomes”. To achieve this, healthcare must be “safe, effective, timely, equitable and patient-centered” [3, 4]. Generally, the quality of healthcare has increased between 2000 and 2015 but it was not equal [5]. Sex, race, and socioeconomic disparities in incidence and mortality of CRC might be attributable to various risk factors like unhealthy diet and physical inactivity, or lack of easy access to screening and early diagnostic modalities or effective treatment resources, which need to be determined and be addressed around the world properly [6–8]. Also, evaluating the inequity in different countries would provide some insights for policymakers to be used in countries with low-quality care. There are indicators such as the socio-demographic index (SDI) to assess the range of development based on the five above-mentioned WHO healthcare criteria. Some initiatives like the World Bank Delivery Indicators and the WHO Global Health Observatory are trying to identify indices to assess the improvements in the quality of care; while, they have not been introduced as comprehensive indices yet [9, 10].

Some indices like the concentration index and horizontal inequity have been introduced to compare inequities in health systems; however, there is not any comprehensive and unbiased index to make the quality of care and inequities comparable [11, 12]. Herein, a new comprehensive index called the quality of care index (QCI) is used to compare the inequities in the quality of care for CRC globally, regionally, and nationally in both sexes and different age groups from 1990 to 2019.

## Methods

### Overview

The methodological details of the Global Burden of Disease (GBD) data have been expressed elsewhere [13–15]. Briefly, the Institute for Health Metrics and Evaluation (IHME) coordinates the GBD study that provides the mortality, incidence, prevalence, years of life lost (YLLs), years lived with disability (YLDs), DALYs, and other measures of 369 diseases and injuries and 87 risk factors in 204 countries and territories from 1990 to 2019 [13, 14]. This study is in accordance with the Guidelines for Accurate and Transparent Health Estimates Reporting (GATHER) [16].

### Data sources

According to the 10th revision of the International Classification of Diseases (ICD), C18-C21.9 (C18: Malignant neoplasm of the colon; C19: Malignant neoplasm of rectosigmoid junction; C20: Malignant neoplasm of the rectum; C21: Malignant neoplasm of anus and anal canal), D01.0-D01.3 (D01.0: Carcinoma in situ: Colon; D01.1: Carcinoma in situ: Rectosigmoid junction; D01.2: Carcinoma in situ: Rectum; D01.3: Carcinoma in situ: Anus and anal canal), D12-D12.9 (Benign neoplasm of colon, rectum, anus and anal canal), and D37.3-D37.5 (D37.3: Neoplasm of uncertain or unknown behavior: Appendix; D37.4: Neoplasm of uncertain or unknown behavior: Colon; D37.5: Neoplasm of uncertain or unknown behavior: Rectum) codes were selected as CRC death, which was mapped to B.1.6 GBD code as CRC [13, 17]. In addition, ICD-10 codes of C18-C19.0, C20, C21-C21.8, Z12.1-Z12.13 (Z12.1: Special screening examination for neoplasm of the intestinal tract), Z85.03-Z85.048 (Z85.0: Personal history of malignant neoplasm of digestive organs), and Z86.010 (Z86.0: Personal history of other neoplasms) were mapped to define CRC new cases [18].

Here, we focus on the QCI to show the inequity in the quality of CRC care by using GBD 1990–2019 data [19]. The details of utilized methods have been explained somewhere else [20]. In brief, we used four stages in order to compute QCI, which were data acquisition (e.g. retrieving data from GBD, retrieving primary measures like DALY, YLL, YLD, death, incidence, prevalence, and manually inspection of each measure for zero estimates), data curation (this step was optional and aimed to reduce the size of the retrieved data), data analysis (i.e. secondary measures calculation and implementation of the principal component analysis (PCA)), and data visualization.

### QCI

The incidence, mortality, prevalence, YLL, YLD, and DALY age-standardized rates were extracted from the GBD study. The mentioned rates and indices were transformed to four ratios, including mortality-to-incidence (MIR), prevalence-to-incidence, DALY-to-prevalence, and YLL-to-YLD. The MIR represents that with considering a stable incidence of CRC, a higher number of mortality represents worse conditions. The prevalence-to-incidence ratio represents that with a similar number of incidence, a higher prevalence shows better management which prevents death. The DALY-to-prevalence ratio indicates that with the same prevalence between regions, higher DALY is representative of the worse quality of care. Finally, the YLL-to-YLD ratio shows the role of the health system in postponing deaths, and higher values shows worse conditions. Then, these measures were combined by the PCA method to create a comprehensive index called QCI that represents the overall quality of care as diagnostic and therapeutic services. PCA is a statistical technique that represents the linear correlation of multiple variables as orthogonal components [21]. The first component of PCA, which had the highest score, was chosen as QCI. The score of QCI was then transformed to a range from zero to 100 using the following formula:

$$QCI\;(x)=\frac{\left[PCA_{score}\;(x)–min\;PCA_{score}\right]}{\left[max\;PCA_{score}–min\;PCA_{score}\right)}$$

Where x is the data point. Higher amounts of QCI represent higher levels of quality of care. Further details on the PCA statistical technique and QCI calculation are provided in S1 Appendix.

### Validation of QCI

The article by Lozano et al. [22] introduced an index called the universal health coverage (UHC) to evaluate the quality of healthcare and to measure the health profiles of countries across the world. The GBD 2019 mapped 23 effective coverage indicators based on types of health services and population age groups for each combination of location and year to measure UHC. Compared with UHC, QCI can be estimated by sex and age and is more convenient for calculation since it only uses basic epidemiological measures, including mortality, incidence, prevalence, YLL, YLD, and DALY instead of using 23 effective coverage indicators and could be estimated for all causes included in the GBD study.

Assessing the results of our developed index showed that it is comparable with UHC. We performed a mixed-effect model analysis on age-standardized values to estimate the correlation between UHC index for CRC treatment and QCI. We considered inpatient admissions and outpatient visits per capita, in addition to attributable burden to CRC risk factors, deaths, and prevalence of CRC as independent variables with fixed effects and considering countries as a random effect, and QCI as a dependent variable [23]. The Pearson correlation coefficient between the predicted QCI obtained from a mixed-effect regression model for CRC and UHC index for CRC treatment was 0.87, representing a significant association between UHC and QCI, which proved the validation of QCI for measurement of quality of care in patients with CRC. The utilized formula for this correlation is provided in S1 Appendix.

### Gender disparity ratio (GDR)

The QCI scores in females were divided by the scores in males and the result was called GDR. The values that were closer to one show less inequity between males and females. Values more than one represented the better quality of care for females, and values less than one indicated better quality of care in males with CRC.

### SDI stratification

Measures of income per capita, total fertility rate, and educational access are included in the SDI definition [24]. Countries and regions are comparable for income and educational levels by SDI. Regions were divided into five quintiles, including high, high-middle, middle, low-middle, and low based on the estimated SDI values [25].

### Statistical analysis

Analyses were conducted and tables and figures were drawn using the R version 3.6.0 and R-studio statistical software for Windows software package (http://www.r-project.org/, RRID: SCR_001905, Vienna, Austria) [26]. Primary measures, including incidence, death, prevalence, YLL, YLD, and DALY rates were reported in 95% uncertainty intervals (UIs) to provide both the interval and point estimations. Uncertainty was determined by sampling 1000 draws at each computational step. Then, uncertainty from multiple sources such as input data, corrections of measurement error, and estimates of residual non-sampling error was combined. UIs were defined as the 25th and 975th values of the ordered draws. The age-standardizing technique was used to make the primary measures results comparable between different locations.

## Results

### Overview

There were 2,166,168 (95% UI: 1,996,298–2,342,842) new cases of CRC in 2019 and it was the cause of death in 1,085,797 (1,002,795–1,149,679) individuals in the same year, globally. The CRC led to 24,284,087 (22,614,920–25,723,221) DALYs in 2019 in comparison with 12,408,147 (11,858,132–12,924,930) in 1990 around the world (Table 1).

**Table 1:** The all-ages numbers and age-standardized rates of incidence, death, and disability-adjusted life years (DALYs) in males, females, and both sexes globally in 1990 and 2019.

	1990						2019					
	Incidence		Deaths		DALYs		Incidence		Deaths		DALYs	
Sex	Number	Rate	Number	Rate	Number	Rate	Number	Rate	Number	Rate	Number	Rate
**Both**	842098 (810408 to 868574)	22.2 (21.3 to 23)	518126 (493682 to 537877)	14.3 (13.5 to 14.9)	12408147 (11858132 to 12924930)	308.5 (294.7 to 320.8)	2166168 (1996298 to 2342842)	26.7 (24.6 to 28.9)	1085797 (1002795 to 1149679)	13.7 (12.6 to 14.5)	24284087 (22614920 to 25723221)	295.5 (275.2 to 313)
**Female**	413893 (393399 to 432693)	19.9 (18.8 to 20.8)	260816 (244563 to 275303)	12.9 (12 to 13.6)	5934541 (5614690 to 6295856)	276.8 (262 to 293.2)	926433 (831857 to 1011626)	21.2 (19 to 23.2)	491622 (437555 to 532378)	11.2 (10 to 12.2)	10324496 (9494907 to 11149967)	237.9 (218.7 to 257.1)
**Male**	428206 (413478 to 444287)	25.2 (24.2 to 26.1)	257310 (246262 to 271018)	16.2 (15.4 to 16.9)	6473606 (6165745 to 6879920)	346.6 (330.9 to 366.7)	1239735 (1133166 to 1359150)	33.1 (30.2 to 36.2)	594176 (550959 to 638031)	16.6 (15.4 to 17.9)	13959591 (12895721 to 15045718)	360.1 (333.1 to 387.8)

Data in parentheses are 95% uncertainty intervals.

Abbreviation: DALYs = disability-adjusted life years.

The age-standardized incidence rate (ASIR) of CRC has increased in all SDI quintiles over 1990–2019, but it barely increased in the highest quintile. The high and low SDI quintiles with 42.8 (38.7–46.6) and 7.3 (6.5–8.1) had the highest and lowest ASIR in 2019, respectively. The age-standardized mortality rate (ASMR) increased in all SDI countries except for the high SDI quintile which decreased from 21.2 (20.1–21.7) in 1990 to 16.3 (14.9–17.1) in 2019. Of note, it barely increased in the high-middle SDI quintile (S1 Table).

In 2019, the European Region and African Region with DALY rates of 386.2 (363.4–407.7) and 190.5 (167.3–215) had the highest and lowest values among WHO regions in both sexes, respectively (S2 Table). Among the 21 GBD regions, Central Europe with 512.6 (448.7–577.9) age-standardized DALYs and 23.6 (20.8–26.4) age-standardized deaths per 100,000 had the highest rates of these measures of CRC among both sexes combined in 2019 (S3 Table).

### QCI

**Fig 1:**
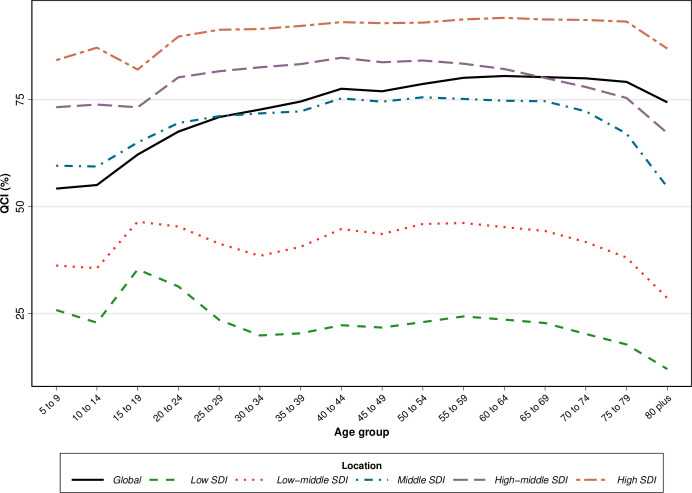
The quality of care index (QCI) of colorectal cancer in both sexes by age and socio-demographic index (SDI) quintiles, 2019.

The QCI score increased from 63.6 in 1990 to 77.6 in 2019 globally (S4 Table). In 2019, the QCI values had a higher difference between females and males in comparison with QCI values in 1990 (78.6 vs. 76.2 in 2019 and 63.9 vs. 63.5 in 1990 in males vs. females, respectively) (S5 Table). QCI values elevated with an increase in age up to 75 years old and started decreasing after this age (Fig 1).

The QCI scores increased in all WHO regions over 1990–2019 and Western Pacific Region had the greatest increase within this period. In 2019, the African Region with a QCI value equal to 23.6 had the lowest QCI value among WHO regions, while the Region of the Americas (84.4) and Western Pacific Region (84.3) had the highest QCI values (S4 Table).

Similar to WHO regions, QCI values in all SDI quintiles had an increase in 2019 compared to 1990, except for the high SDI quintile which had a fairly substantial decrease and the high-middle SDI quintile which had a slight decrease in this regard. Also, there was a positive association between SDI quintiles and QCI values in which more developed countries had higher QCI values. In the low and low-middle SDI quintile, there was a peak in QCI in the 15–19 age group, whereas high and high-middle SDI countries showed a remarkable decrease in this age group in 2019 (Fig 1).

**Fig 2:**
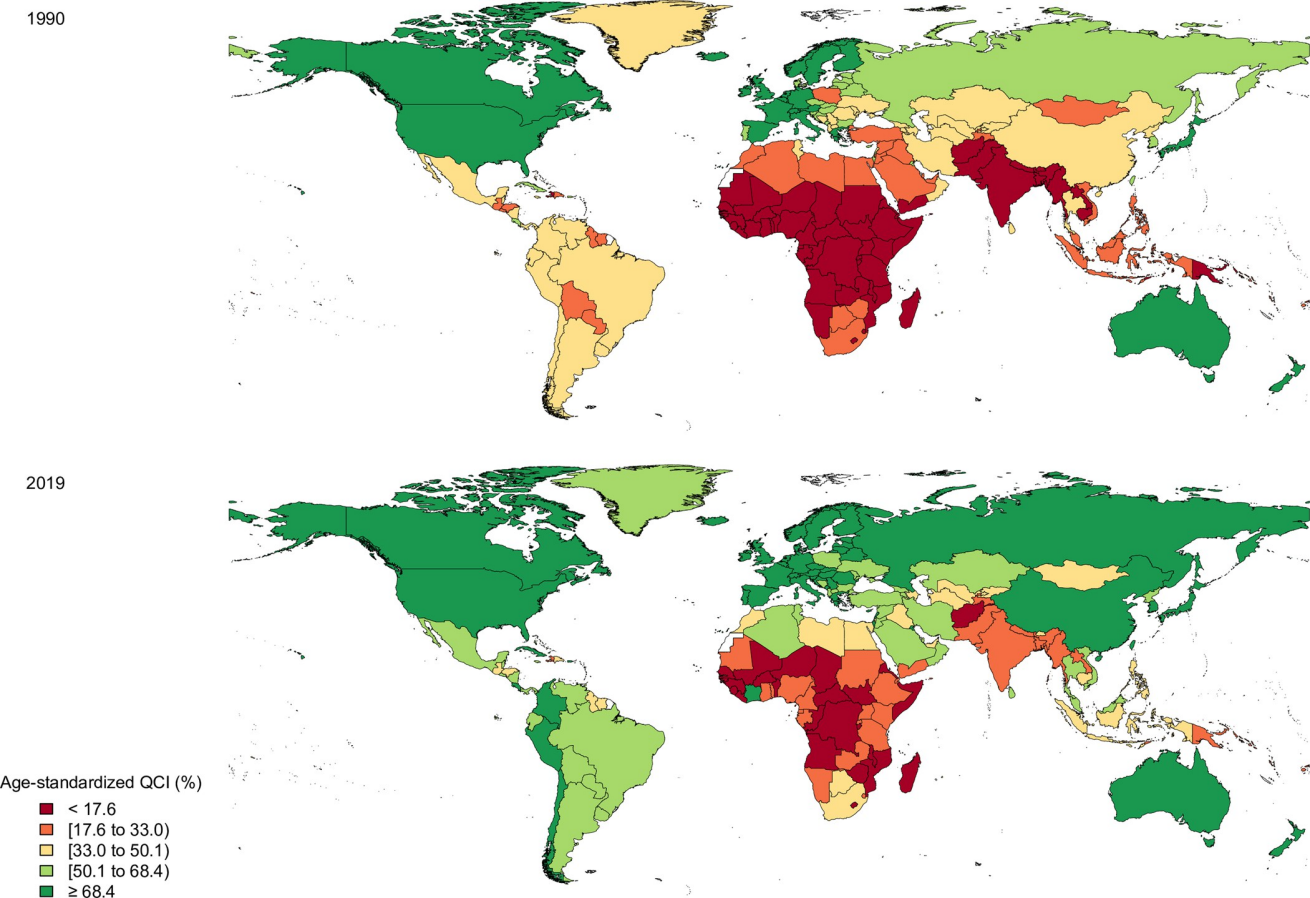
The quality of care index (QCI) of colorectal cancer in both sexes by country in 1990 and 2019. Contains information from OpenStreetMap and OpenStreetMap Foundation, which is made available under the Open Database License.

In 1990, Eritrea and the United States of America with QCI values of 0.9 and 87.3 had the lowest and highest quality of care among both sexes, respectively (Fig 2 and S4 Table). In 2019, Japan (98.2), Australia (97.8), and Canada (97.5) had the greatest quality of care in the world, while the Central African Republic (4.6), Somalia (7.3), and South Sudan (9.6) had the lowest QCI values (Fig 2 and S4 Table). By sex, Japan (87.8) and Eritrea (0.0) had the highest and lowest QCI values in males and Switzerland (89.5) and Afghanistan (0.3) had the highest and lowest QCI in females in 1990, respectively (S5 Table and S1 Fig). In 2019, the highest quality of care for males was in Japan (98.7) and Canada (97.7), whereas the Central African Republic (5.2) and Somalia (8.9) had the lowest quality of care. For females, Switzerland (99.1) and Australia (97.7) had the highest, while Afghanistan (5.3) and the Central African Republic (6.3) had the lowest QCI values in 2019 (S5 Table and S2 Fig).

### Gender and age disparity

**Fig 3:**
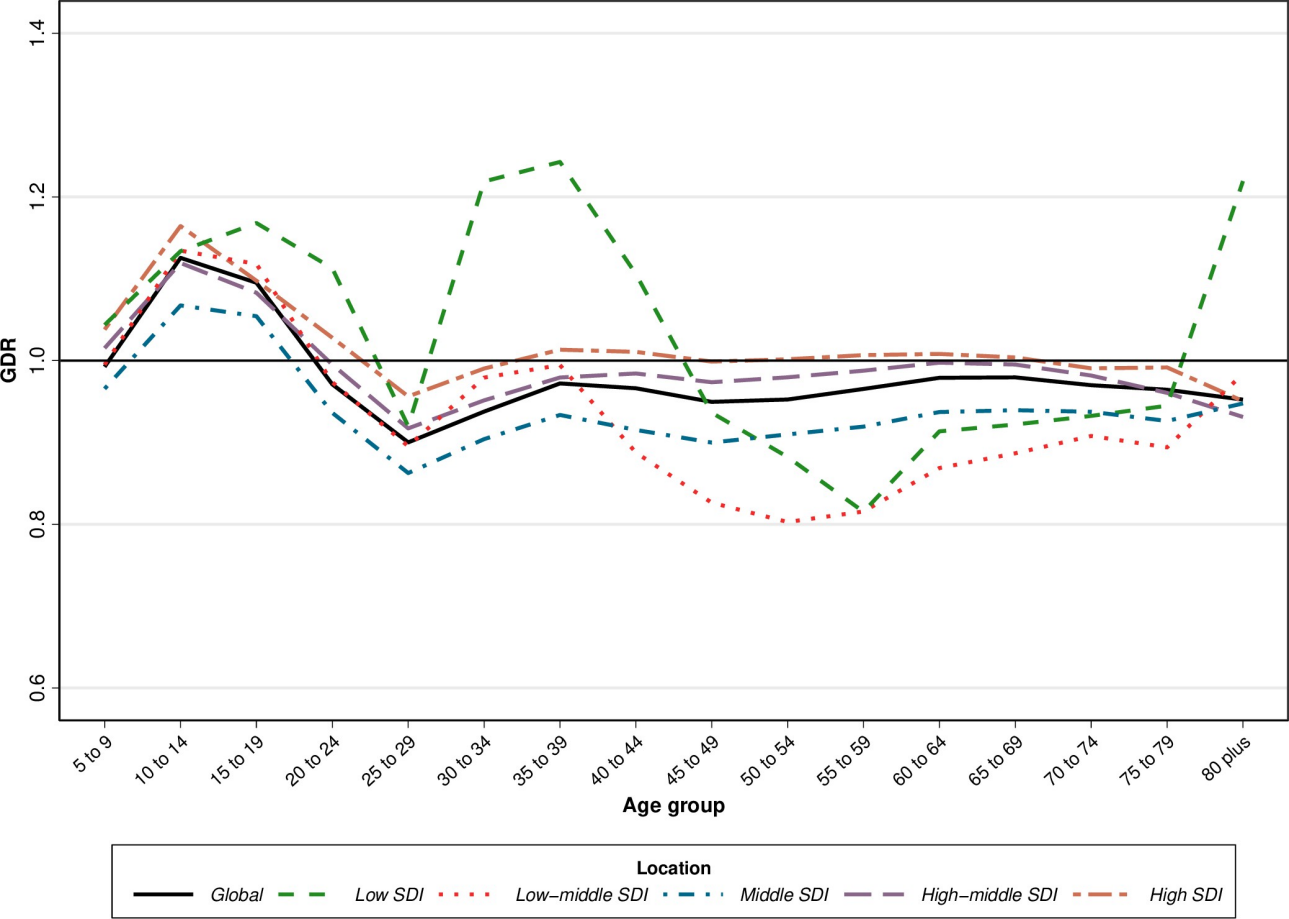
The gender disparity ratio (GDR) of colorectal cancer in both sexes by age and socio-demographic index (SDI) quintiles, 2019.

The GDR values were 1.0 in 1990 and 2019 worldwide. In 2019, all WHO regions had GDR equal to one other than the Eastern Mediterranean Region and African Region. Also, Eastern Mediterranean Region had the lowest GDR in 1990 and 2019 (0.6 and 0.7 in 1990 and 2019, respectively) (S5 Table). Globally, the GDR had the highest values in 10–14 years old and was higher than one up to 20 years old, while it had the lowest value in 25–29 age group and was lower than one then after, which represented a better quality of care for males in 2019 (Fig 3).

The GDR values in high and high-middle SDI quintiles were 1.0 in 1990 and 2019. Middle and low SDI quintiles had a GDR of 0.9 in 1990 and 1.0 in 2019. The low-middle SDI quintile had GDR values of 0.9 in both 1990 and 2019. The GDR in low and middle SDI quintiles increased from 0.9 to 1.0 from 1990 to 2019 (S5 Table). The age trend of GDR among SDI quintiles in 2019 was almost similar to the global trend except for the low SDI quintile that peaked at 35–39 years old and had the lowest values in 55–59 age group and low-middle SDI that the lowest GDR values were noted in 50–54 years old (Fig 3).

**Fig 4:**
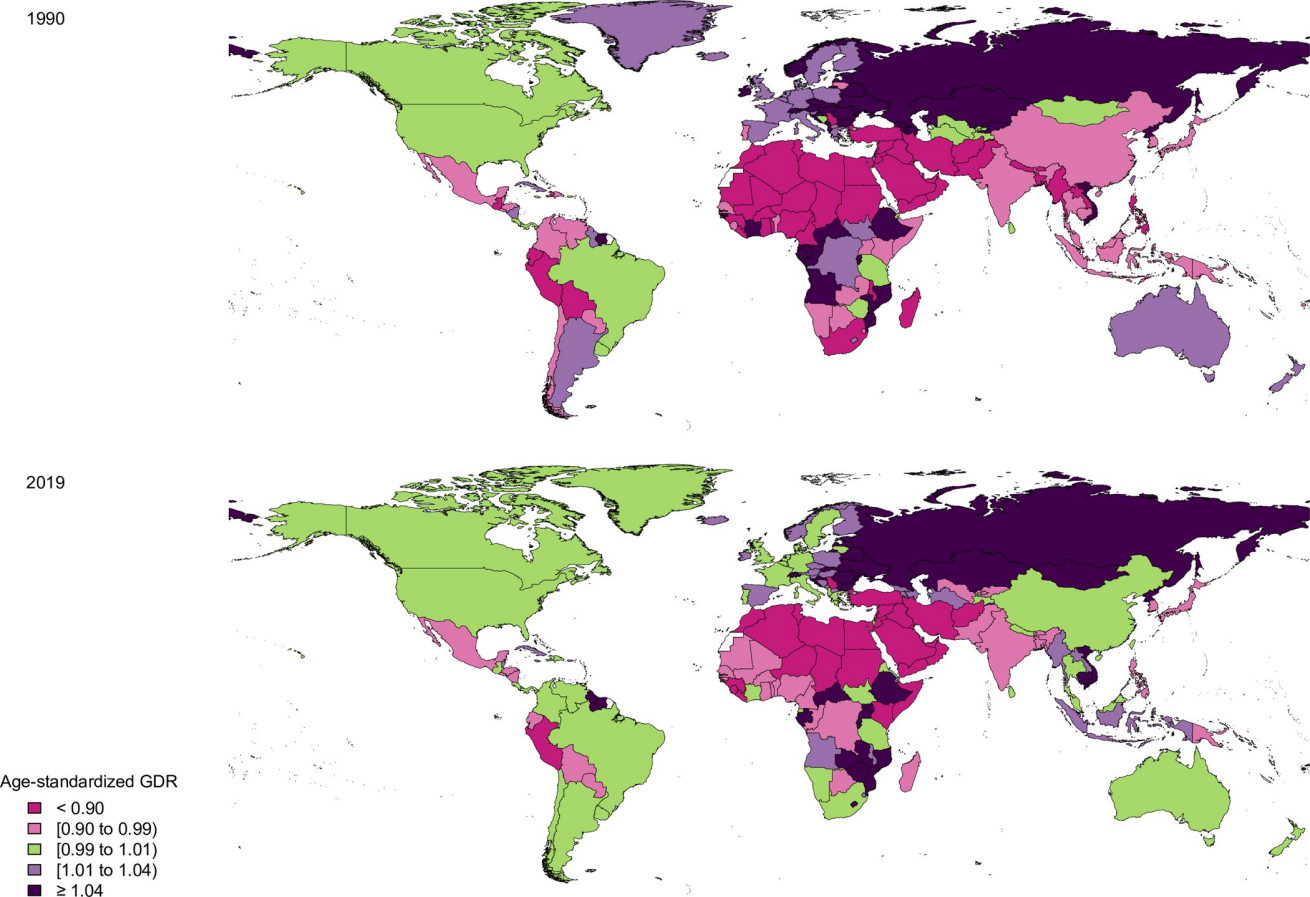
The gender disparity ratio (GDR) of colorectal cancer in both sexes by country in 1990 and 2019. Contains information from OpenStreetMap and OpenStreetMap Foundation, which is made available under the Open Database License.

In 1990, Central African Republic (2.5), Equatorial Guinea (2.1), and Burundi (1.4) had the highest GDR values, however, Afghanistan (0.0), Saudi Arabia (0.2), Yemen (0.5), and Sudan (0.5) had the lowest values (Fig 4 and S5 Table). In 2019, the Central African Republic (1.2), Russian Federation (1.2), and Ukraine (1.2) had the highest, while Afghanistan (0.3), Yemen (0.6), Morocco (0.6), Sudan (0.6), and Egypt (0.6) had the lowest GDR values (Fig 4 and S5 Table).

## Discussion

In the QCI estimation for different causes, we provided the quality of care for CRC in the present study. Previous works provided QCI for hematologic malignancies, thyroid cancer, pancreatic cancer, brain and other central nervous system cancers, and ischemic heart disease [27–31]. Findings of our developed index showed that the QCI values have increased within the last three decades. However, there is still a major gap between high and low SDI quintiles. Also, the quality of care in African and South-East Asia Regions was at the lowest levels and there was still gender inequity in some WHO regions, especially in Eastern Mediterranean Region. The global trend of QCI in most age groups was almost increased for up to the age of 75, and it had a high level of inequities in different SDI quintiles. Comparing the gender inequity showed that the global GDR for above 20-year-old individuals was in favor of males.

The reduction in QCI values was initiated in individuals below 70 years old in all SDI quintiles except for the high SDI quintile which started in individuals 75 years old. This finding might be due to the recommended screening strategies by the United States Preventive Service Task Force (USPSTF). The USPSTF recommends screening strategies like fecal occult blood test (FOBT) and total colonoscopy for up to 75-year-old individuals and it does not recommend for 85 or older people, so it might explain the reason of the decreasing QCI scores in older age groups [32]. Moreover, in some other high SDI countries like Germany, Switzerland, Norway, Sweden, Canada, and the Netherlands, the screening interventions, especially fecal immunochemical test (FIT) are recommended for people between 50 and 75 years old [33]. The mentioned reduction was steeper in middle and high-middle SDI quintiles. The prevention strategies implemented in Japan, the first rank of QCI, was immunochemical-FOBT in individuals aged 40 years old [34]. As the incidence rate of CRC in high SDI countries is higher, the screening programs and diagnostic and treatment methods are more accessible. Therefore, the QCI values were higher than the values in the low SDI quintile [35, 36].

A cross-sectional study conducted in five Spanish regions aimed to investigate the age disparities in presentations, diagnosis, and treatment strategies for patients with CRC in <65, 65–79, and ≥80 years old age groups, showed prominent differences in help-seeking behaviors and treatment characteristics between the age groups, while no variation was revealed in primary and secondary healthcare investigations [37]. In this regard, a study by Serra-Rexach et al. on 503 patients with CRC divided into two groups, young group and older group (≥75 years old), showed that young patients were more likely to receive surgery (P-value = 0.005), radiotherapy (P-value = 0.0001), and chemotherapy (P-value = 0.001), while older individuals received higher levels of palliative cares (P-value = 0.004). Also, tumor-specific mortality rate of young group was lower (hazard ratio (HR) = 0.66; 95% confidence interval (CI): 0.45–0.97) [38]. Our results also showed a slight decrease in QCI values from the age of 75 which might be due to the under-treatment in this age group according to the previously mentioned studies.

A prospective cohort study on 403 patients with CRC showed that males had an increased probability of readmission to hospitals than females (HR = 1.52; 95% CI: 1.17–1.96) [39]. We represented that regions and countries with lower QCI scores like Eastern Mediterranean Region and African Region in addition to Afghanistan country also had GDR scores in favor of males. There are various factors like biological variations, socioeconomic status, race/ethnicity, and culture in determining equity in access to healthcare services for cancers [40]. As a result, the differences in quality of care in patients with CRC between males and females can be explained by mentioned factors.

To prevent the incident cases of CRC, 40-year-old or older people should be screened annually by FOBT that is a noninvasive method or colonoscopy every 10 years which is cost-effectiveness [41]. According to a national survey in Japan, some promotion strategies like personal and household invitation letters and home visits are more effective strategies to encourage people, especially in the 60–69 age group, rather than free cancer screening programs and screening in medical offices [42]. With improved screening the prevalent cases would be increases and as a result, the incidence rates would increase and the mortality and DALY rates would decrease [43]. Smoking is a major risk factor of CRC [44]. Moreover, there are different measures at the population level like increasing the cost of cigarettes, facilitation of pharmacologic and behavioral therapies regarding smoking cessation, and anti-tobacco campaigns to reduce the prevalence of smoking [45]. Some prevention strategies like flexible sigmoidoscopy can be used in low SDI quintiles, and it can be combined with the FIT if applicable [46]. Kingsley J. et al. showed that screening colonoscopy is not a cost-effective method in comparison with FIT as long as the inadequate bowel preparation is higher than 13% [47]. Also, it is more invasive and more expensive than other common screening strategies [48]. As a result, populations living in regions with lower SDIs might not have access to or afford the screening programs [49, 50]. Also, Gancayco et al. showed that utilizing screening colonoscopy decreased with advancing age [51].

In the UHC measurement framework, 23 effective coverage indicators covering five health service domains including promotion, prevention, treatment (i.e. communicable diseases, maternal, neonatal, and child health and non-communicable diseases), rehabilitation, and palliation) and five-population age groups (i.e. reproductive and newborn, children younger than 5 years, children and adolescents aged 5–19 years, adults aged 20–64 years, and older adults aged ≥65 years were included [22]. The overall goal of UHC effective coverage was to estimate the quality care access, whereas our developed index aimed to represent the inequity in the quality of care based on GBD 2019 study for 369 diseases and injuries in 204 countries and territories from 1990 to 2019. Also, out of 23 indicators, 19 were mortality-based measures which were generated by MIRs and mortality-to-prevalence ratios, and the UHC effective coverage was constructed by weighting each effective coverage indicator based on potential health gains [22]. However, we just used four ratios which were combined by PCA. Nevertheless, both measures have been transformed to a scale of 0–100 values, in a way that higher values show better conditions. Also, another advantage of our developed index compared with the UHC is that QCI can be estimated by sex, age groups, cause, and is available for each year between 1990 and 2019, while the UHC index is currently available for only 1990, 2010, and 2019 and has not been evaluated by sex and age groups.

Developing the QCI to make the comparison of quality of care and to generate GDR are strengths of the study. However, this study has some limitations. First, the GBD has not categorized the population based on ethnicity, so racial inequity could not be compared. Second, various types of CRC such as mucinous, signet cell ring, medullary, micropapillary, serrated, undifferentiated and their histopathologic features like microsatellite instability status that is one of the exterminators of management strategies were not included in this study [52]. Third, the ICD-10 codes in the CRC definition seem overly broad (inclusion of anal cancers beyond C21.8; carcinoma in situ, including that of anus; D37.3: neoplasm of uncertain or unknown behavior; and Z codes for healthcare exams), so it might lead to overestimation of the results. It should be taken into consideration that most of the limitations were due to GBD methods for data gathering and reporting, so we could not manipulate them. Despite these limitations, the GBD study is one of the most comprehensive and up-to-date projects that meter and evaluate the burden of diseases.

## Conclusion

The ASIR trend of CRC despite its ASMR was increasing, which can represent that therapeutic interventions regarding CRC have been more effective or more available than preventive measures. The low and low-middle SDI quintiles and African Region shall make efficient policies to improve the quality of care, especially for women. Our findings on the interpretation of QCI and GDR could be used by global, regional, and national health policymakers to prepare equitable care for patients suffering from CRC worldwide. Further studies might be needed to evaluate the implementation of this quality measure and to develop quality measurement systems to improve and effectively monitor cancer care.

## Supporting information

S1 Table The incidence, death, and disability-adjusted life years (DALYs) numbers and age-standardized rates in socio-demographic index (SDI) quintile in males, females, and both sexes in 1990 and 2019. (DOCX)

S2 Table The incidence, death, and disability-adjusted life years (DALYs) numbers and age-standardized rates in the World Health Organization Regions in males, females, and both sexes in 1990 and 2019. (DOCX)

S3 Table The incidence, death, and disability-adjusted life years (DALYs) numbers and age-standardized rates in 21 Global Burden of Disease (GBD) regions in males, females, and both sexes in 1990 and 2019. (DOCX)

S4 Table Quality of care index (QCI) in the world, World Health Organization Regions, socio-demographic index (SDI) quintiles, and countries in both sexes in 1990 and 2019. (DOCX)

S5 Table Gender disparity ratio (GDR) values in the world, World Health Organization Regions, socio-demographic index (SDI) quintiles, and countries by sex in 1990 and 2019. (DOCX)

S1 Fig The quality of care index (QCI) of colorectal cancer in females and males by country in 1990. (PDF)

S2 Fig The quality of care index (QCI) of colorectal cancer in females and males by country in 2019. (PDF)

S1 Appendix Details of mathematical calculation of quality of care index. (DOCX)

## List of abbreviations

CRC: Colorectal cancer
DALYs: Disability-adjusted life years
WHO: World Health Organization
SDI: Socio-demographic index
QCI: Quality of care index
GBD: Global Burden of Disease
IHME: Institute for Health Metrics and Evaluation
YLLs: Years of life lost
YLDs: Years lived with disability
GATHER: Guidelines for Accurate and Transparent Health Estimates Reporting
ICD: International Classification of Diseases
MIR: Mortality-to-incidence ratio
PCA: Principal component analysis
UHC: Universal health coverage
GDR: Gender disparity ratio
UI: Uncertainty interval
USPSTF: US Preventive Service Task Force
FOBT: Fecal occult blood test
FIT: Fecal immunochemical test
HR: Hazard ratio
CI: Confidence interval
OR: Odds ratio
