# Supplementary material for: The global, regional, and national burden and quality of care index (QCI) of colorectal cancer; a global burden of disease systematic analysis 1990–2019
Source: PLoS One. 2022 Apr 21;17(4):e0263403. doi: 10.1371/journal.pone.0263403 (PMC9022854; doi:10.1371/journal.pone.0263403)
Supplement: S1 Table — (DOCX) [file pone.0263403.s001.docx]

**S1 Table.** The incidence, deaths, and disability-adjusted life years (DALYs) numbers and age-standardized rates in socio-demographic index (SDI) quintile in males, females, and both sexes in 1990 and 2019

|  | | **1990** | | | | | | **2019** | | | | | |
| --- | --- | --- | --- | --- | --- | --- | --- | --- | --- | --- | --- | --- | --- |
|  |  | **Incidence** | | **Deaths** | | **DALYs** | | **Incidence** | | **Deaths** | | **DALYs** | |
| **Location** | **Sex** | **Number** | **Rate** | **Number** | **Rate** | **Number** | **Rate** | **Number** | **Rate** | **Number** | **Rate** | **Number** | **Rate** |
| **High SDI** | **Both** | 443895 (427714 to 453338) | 42.5 (40.9 to 43.4) | 222296 (211298 to 228215) | 21.2 (20.1 to 21.7) | 4660354 (4514653 to 4766630) | 454.7 (441.1 to 465.1) | 798580 (715631 to 873249) | 42.8 (38.7 to 46.6) | 327570 (294904 to 345578) | 16.3 (14.9 to 17.1) | 6164656 (5754647 to 6435898) | 347.3 (328.2 to 361.6) |
|  | **Female** | 216161 (204534 to 222963) | 35.4 (33.7 to 36.4) | 112870 (105091 to 117168) | 17.8 (16.7 to 18.5) | 2196296 (2093658 to 2259320) | 377.7 (362.7 to 387.7) | 351798 (304599 to 389395) | 34.1 (30.1 to 37.5) | 154370 (132931 to 166387) | 13.1 (11.7 to 14) | 2648358 (2413265 to 2809117) | 275.2 (256.6 to 289) |
|  | **Male** | 227734 (222100 to 231776) | 52.1 (50.6 to 53.1) | 109426 (105937 to 111493) | 26 (25 to 26.6) | 2464058 (2404537 to 2512193) | 554.8 (541.1 to 566) | 446781 (403357 to 492764) | 52.8 (47.7 to 58.3) | 173200 (161438 to 180974) | 20.1 (18.8 to 21) | 3516298 (3349007 to 3660216) | 428.1 (408.7 to 445.3) |
| **High-middle SDI** | **Both** | 237898 (229789 to 246091) | 22.6 (21.8 to 23.4) | 162685 (155451 to 168785) | 16.1 (15.3 to 16.7) | 3960497 (3798199 to 4115133) | 364.4 (349.2 to 378.4) | 655822 (594856 to 716673) | 32.4 (29.4 to 35.4) | 326640 (299662 to 349530) | 16.2 (14.9 to 17.4) | 7174858 (6649069 to 7693263) | 356.6 (330.6 to 382.6) |
|  | **Female** | 120445 (114909 to 126417) | 20.1 (19.1 to 21.1) | 83273 (78839 to 87590) | 14.1 (13.3 to 14.9) | 1931150 (1833990 to 2036665) | 320.9 (304.6 to 338.5) | 274507 (243759 to 304485) | 24.6 (21.8 to 27.3) | 144781 (130329 to 157757) | 12.6 (11.4 to 13.7) | 2967718 (2714971 to 3225452) | 272.1 (249 to 296.3) |
|  | **Male** | 117453 (112447 to 122523) | 26.3 (25.2 to 27.4) | 79413 (75877 to 82999) | 19.1 (18.2 to 19.9) | 2029347 (1927870 to 2138829) | 423.3 (403.6 to 444.1) | 381315 (340262 to 428501) | 42.1 (37.6 to 47.2) | 181860 (165132 to 197727) | 21.1 (19.2 to 22.9) | 4207141 (3811184 to 4603306) | 457.3 (414.2 to 498.6) |
| **Middle SDI** | **Both** | 104834 (97278 to 113063) | 10.2 (9.5 to 11) | 83375 (76841 to 90067) | 8.8 (8.1 to 9.4) | 2365882 (2176493 to 2557301) | 208.2 (191.8 to 224.7) | 465980 (418598 to 515360) | 18.9 (17 to 20.9) | 279778 (251149 to 306137) | 12 (10.8 to 13.1) | 6990430 (6308897 to 7671286) | 273.6 (247 to 299.9) |
|  | **Female** | 50553 (45520 to 55852) | 9.6 (8.7 to 10.6) | 40776 (36934 to 45254) | 8.3 (7.5 to 9.1) | 1119879 (1005009 to 1248541) | 195.6 (176.8 to 217.3) | 207924 (181058 to 236631) | 16.2 (14.1 to 18.4) | 118328 (103747 to 133323) | 9.6 (8.4 to 10.8) | 2804249 (2477310 to 3159893) | 213.2 (188.4 to 240.1) |
|  | **Male** | 54281 (48799 to 60378) | 10.9 (9.8 to 12) | 42599 (38425 to 47396) | 9.3 (8.5 to 10.3) | 1246003 (1120068 to 1392094) | 221.7 (200 to 247) | 258055 (227291 to 295939) | 21.8 (19.2 to 24.8) | 161450 (141994 to 182814) | 14.6 (12.9 to 16.5) | 4186181 (3674378 to 4729310) | 338.6 (298.4 to 381.6) |
| **Low-middle SDI** | **Both** | 40778 (36760 to 45652) | 6.9 (6.2 to 7.7) | 36051 (32664 to 40412) | 6.5 (5.9 to 7.3) | 1034041 (932274 to 1157750) | 155.9 (141.3 to 174.8) | 151948 (137798 to 166926) | 11.3 (10.2 to 12.4) | 116546 (105505 to 128325) | 9.1 (8.2 to 9.9) | 2998329 (2703935 to 3314970) | 209.5 (189.4 to 231.1) |
|  | **Female** | 20023 (17278 to 23339) | 6.8 (5.9 to 7.9) | 17678 (15242 to 20649) | 6.4 (5.5 to 7.4) | 508018 (433935 to 596982) | 154.6 (133 to 180.7) | 72919 (64020 to 82556) | 10.4 (9.1 to 11.7) | 57152 (49649 to 64902) | 8.5 (7.4 to 9.6) | 1444687 (1245771 to 1646536) | 195.6 (169.1 to 223.2) |
|  | **Male** | 20755 (18469 to 25410) | 6.9 (6.2 to 8.4) | 18373 (16276 to 22615) | 6.6 (5.9 to 8) | 526023 (467456 to 650876) | 157.1 (139.4 to 193.7) | 79030 (70594 to 87627) | 12.2 (10.9 to 13.5) | 59395 (53161 to 66174) | 9.7 (8.7 to 10.8) | 1553642 (1385338 to 1735400) | 224.6 (200.8 to 250.7) |
| **Low SDI** | **Both** | 14278 (11962 to 16828) | 6.2 (5.2 to 7.3) | 13443 (11307 to 15790) | 6.2 (5.2 to 7.3) | 381057 (318699 to 450051) | 146.2 (123 to 172.2) | 36948 (32893 to 41032) | 7.3 (6.5 to 8.1) | 34656 (30959 to 38606) | 7.3 (6.5 to 8.1) | 942417 (835787 to 1059266) | 166.1 (148.4 to 185.9) |
|  | **Female** | 6501 (5010 to 8330) | 5.7 (4.4 to 7.2) | 6078 (4741 to 7834) | 5.7 (4.4 to 7.2) | 176089 (135439 to 228769) | 135.1 (105.1 to 174.5) | 18781 (16514 to 21163) | 7.3 (6.4 to 8.2) | 16696 (14625 to 18857) | 6.9 (6 to 7.7) | 453326 (394875 to 514850) | 156.4 (136.7 to 176.9) |
|  | **Male** | 7777 (6354 to 9926) | 6.7 (5.5 to 8.4) | 7366 (6039 to 9375) | 6.8 (5.6 to 8.4) | 204968 (168328 to 265266) | 156.8 (128.7 to 200.2) | 18167 (15898 to 20883) | 7.4 (6.5 to 8.5) | 17959 (15618 to 20649) | 7.8 (6.9 to 9) | 489091 (421868 to 569299) | 176.2 (153.4 to 203) |

Data in parentheses are 95% uncertainty intervals

Abbreviations: SDI = socio-demographic index, DALYs = disability-adjusted life years
