# Supplementary material for: The global, regional, and national burden and quality of care index (QCI) of colorectal cancer; a global burden of disease systematic analysis 1990–2019
Source: PLoS One. 2022 Apr 21;17(4):e0263403. doi: 10.1371/journal.pone.0263403 (PMC9022854; doi:10.1371/journal.pone.0263403)
Supplement: S2 Table — (DOCX) [file pone.0263403.s002.docx]

**S2 Table.** The incidence, death, and disability-adjusted life years (DALYs) numbers and age-standardized rates in the World Health Organization Regions in males, females, and both sexes in 1990 and 2019

|  | | **1990** | | | | | | **2019** | | | | | |
| --- | --- | --- | --- | --- | --- | --- | --- | --- | --- | --- | --- | --- | --- |
|  |  | **Incidence** | | **Deaths** | | **DALYs** | | **Incidence** | | **Deaths** | | **DALYs** | |
| **Location** | **Sex** | **Number** | **Rate** | **Number** | **Rate** | **Number** | **Rate** | **Number** | **Rate** | **Number** | **Rate** | **Number** | **Rate** |
| **African Region** | **Both** | 15937 (13532 to 18262) | 7.5 (6.4 to 8.5) | 14843 (12589 to 17051) | 7.4 (6.3 to 8.5) | 399924 (339359 to 463577) | 166.4 (141.3 to 192.2) | 43989 (38772 to 49579) | 9.4 (8.4 to 10.5) | 38200 (33746 to 42945) | 8.8 (7.8 to 9.7) | 1010138 (877000 to 1154199) | 190.5 (167.3 to 215) |
|  | **Female** | 7478 (6198 to 8983) | 6.9 (5.7 to 8.2) | 6994 (5824 to 8402) | 6.9 (5.7 to 8.1) | 184789 (153432 to 225911) | 151.5 (125.5 to 183.2) | 20939 (18316 to 23896) | 8.5 (7.5 to 9.6) | 18498 (16042 to 21044) | 8 (7 to 9) | 476146 (406348 to 551659) | 171.3 (147.3 to 196.5) |
|  | **Male** | 8459 (7038 to 9903) | 8.1 (6.7 to 9.3) | 7848 (6482 to 9182) | 8 (6.7 to 9.2) | 215134 (177476 to 255256) | 181.3 (149.9 to 212.4) | 23050 (19964 to 26316) | 10.4 (9.2 to 11.7) | 19703 (17256 to 22559) | 9.6 (8.6 to 10.9) | 533992 (459227 to 619185) | 211.8 (184.7 to 243.5) |
| **Eastern Mediterranean Region** | **Both** | 12285 (10601 to 14109) | 6.7 (5.8 to 7.7) | 10732 (9259 to 12342) | 6.3 (5.4 to 7.2) | 298564 (258015 to 345580) | 148.8 (128.7 to 171.4) | 48361 (42538 to 55286) | 11.3 (10 to 12.8) | 34085 (29797 to 38883) | 8.7 (7.6 to 9.9) | 945372 (820253 to 1084757) | 199.7 (174.8 to 227.5) |
|  | **Female** | 5461 (4684 to 6392) | 6.3 (5.4 to 7.4) | 4947 (4228 to 5782) | 6.1 (5.2 to 7) | 138361 (119313 to 163676) | 144.2 (123.9 to 169.7) | 19597 (17047 to 22789) | 9.7 (8.4 to 11.2) | 15308 (13206 to 17823) | 8.1 (7 to 9.4) | 423009 (361406 to 495881) | 185.7 (159.9 to 216.7) |
|  | **Male** | 6823 (5698 to 8066) | 7.1 (5.9 to 8.4) | 5786 (4817 to 6814) | 6.4 (5.4 to 7.5) | 160203 (132990 to 189720) | 152.6 (127.3 to 180.2) | 28764 (24786 to 33875) | 12.8 (11.1 to 15) | 18776 (16145 to 22245) | 9.2 (7.9 to 10.8) | 522363 (445338 to 619546) | 212.7 (182.2 to 251.2) |
| **European Region** | **Both** | 353264 (341301 to 361220) | 32.9 (31.8 to 33.7) | 221061 (212191 to 226690) | 20.8 (19.9 to 21.3) | 4809046 (4684269 to 4922857) | 453.9 (441.6 to 465) | 601397 (539749 to 663684) | 37.6 (33.9 to 41.6) | 305815 (280956 to 323735) | 18.2 (16.9 to 19.3) | 5930070 (5565633 to 6267846) | 386.2 (363.4 to 407.7) |
|  | **Female** | 181699 (173420 to 187301) | 28.2 (26.9 to 29) | 116257 (109664 to 120419) | 17.7 (16.7 to 18.3) | 2374414 (2289163 to 2450760) | 384.1 (371.1 to 396.8) | 273797 (242183 to 304195) | 29.8 (26.7 to 33.1) | 145499 (130340 to 155616) | 14.5 (13.2 to 15.5) | 2623345 (2420416 to 2803615) | 303.5 (282.7 to 324.1) |
|  | **Male** | 171565 (167466 to 174938) | 40.5 (39.4 to 41.4) | 104804 (102070 to 106940) | 25.8 (25 to 26.4) | 2434632 (2379446 to 2485184) | 556.2 (542.7 to 568.3) | 327599 (294579 to 363321) | 47.8 (43.1 to 53.1) | 160316 (149065 to 169502) | 23.4 (21.7 to 24.8) | 3306725 (3108359 to 3500325) | 490.4 (460.9 to 519.2) |
| **Region of the Americas** | **Both** | 202742 (195053 to 207633) | 33.3 (32 to 34.1) | 99113 (94095 to 101956) | 16.5 (15.6 to 17) | 2173414 (2106527 to 2227887) | 354.8 (343.8 to 363.8) | 390096 (350940 to 432308) | 30.7 (27.6 to 34) | 175891 (162436 to 184676) | 13.7 (12.7 to 14.3) | 3820607 (3623970 to 4008410) | 305.7 (290 to 320.8) |
|  | **Female** | 100563 (95065 to 103959) | 29.3 (27.8 to 30.2) | 50777 (47450 to 52736) | 14.7 (13.8 to 15.2) | 1052849 (1012083 to 1084176) | 312.4 (300.9 to 321.5) | 183260 (159146 to 207253) | 26.3 (22.9 to 29.8) | 86071 (77570 to 91316) | 11.9 (10.9 to 12.6) | 1760094 (1644990 to 1860761) | 261.7 (245.3 to 276.8) |
|  | **Male** | 102179 (99207 to 104303) | 38.3 (37 to 39.2) | 48336 (46634 to 49458) | 18.8 (18 to 19.3) | 1120565 (1091823 to 1145259) | 406.1 (394.8 to 415.4) | 206836 (180577 to 236968) | 35.7 (31.2 to 40.9) | 89820 (84536 to 93889) | 15.7 (14.8 to 16.4) | 2060513 (1965984 to 2160415) | 355.7 (339.2 to 373.2) |
| **South-East Asia Region** | **Both** | 45996 (40753 to 51342) | 6.8 (6 to 7.6) | 40316 (35702 to 45256) | 6.5 (5.8 to 7.3) | 1174826 (1033147 to 1318941) | 152 (134.5 to 170.2) | 174676 (149850 to 197216) | 10.4 (8.9 to 11.7) | 137666 (119130 to 157365) | 8.6 (7.5 to 9.8) | 3499840 (3027277 to 4006647) | 195.9 (169.7 to 223.5) |
|  | **Female** | 22786 (18843 to 27090) | 6.8 (5.7 to 8) | 19920 (16417 to 23779) | 6.4 (5.4 to 7.7) | 584098 (478228 to 698202) | 152.7 (125.9 to 181.7) | 86562 (70882 to 102589) | 10 (8.2 to 11.7) | 69035 (56629 to 81152) | 8.3 (6.9 to 9.8) | 1723927 (1404047 to 2044779) | 188.5 (153.7 to 223.1) |
|  | **Male** | 23210 (20394 to 28398) | 6.8 (6 to 8.3) | 20397 (17945 to 25121) | 6.5 (5.8 to 7.9) | 590728 (521132 to 727631) | 151.4 (133.8 to 185.8) | 88114 (74495 to 102286) | 10.8 (9.2 to 12.5) | 68631 (58117 to 79824) | 9 (7.6 to 10.4) | 1775913 (1499901 to 2072874) | 203.9 (172.4 to 237.8) |
| **Western Pacific Region** | **Both** | 208385 (194933 to 222655) | 18.5 (17.3 to 19.7) | 129865 (119373 to 140047) | 12.3 (11.3 to 13.2) | 3499000 (3191652 to 3793864) | 283.7 (259.7 to 306.5) | 897401 (798465 to 1008172) | 33.5 (29.8 to 37.5) | 389141 (346912 to 432357) | 14.9 (13.2 to 16.6) | 8974138 (7975836 to 10037591) | 335.2 (298.6 to 374.3) |
|  | **Female** | 94379 (86150 to 103244) | 15.9 (14.5 to 17.3) | 60944 (54521 to 67556) | 10.7 (9.6 to 11.8) | 1577635 (1399151 to 1764212) | 248.9 (221.4 to 277.3) | 337949 (285011 to 393906) | 23.8 (20 to 27.7) | 155022 (130195 to 177257) | 10.8 (9.1 to 12.4) | 3274904 (2821996 to 3745559) | 234.4 (202.1 to 268.5) |
|  | **Male** | 114006 (104384 to 124338) | 21.9 (20.3 to 23.7) | 68921 (61580 to 76708) | 14.5 (13.2 to 15.9) | 1921365 (1707359 to 2158151) | 325.2 (291 to 361.4) | 559452 (478134 to 655585) | 44.5 (38.3 to 52.1) | 234119 (199796 to 271241) | 19.9 (17.2 to 22.8) | 5699234 (4857344 to 6679767) | 445.3 (382.2 to 518.6) |

Data in parentheses are 95% uncertainty intervals

Abbreviations: SDI = socio-demographic index, DALYs = disability-adjusted life years
