# Supplementary material for: The global, regional, and national burden and quality of care index (QCI) of colorectal cancer; a global burden of disease systematic analysis 1990–2019
Source: PLoS One. 2022 Apr 21;17(4):e0263403. doi: 10.1371/journal.pone.0263403 (PMC9022854; doi:10.1371/journal.pone.0263403)
Supplement: S3 Table — (DOCX) [file pone.0263403.s003.docx]

**S3 Table.** The incidence, death, and disability-adjusted life years (DALYs) numbers and age-standardized rates in 21 Global Burden of Disease (GBD) regions in males, females, and both sexes in 1990 and 2019

|  | | **1990** | | | | | | **2019** | | | | | | |
| --- | --- | --- | --- | --- | --- | --- | --- | --- | --- | --- | --- | --- | --- | --- |
|  | | **Incidence** | | **Deaths** | | **DALYs** | | **Incidence** | | **Deaths** | | **DALYs** | | |
| **Location** | **Sex** | **Number** | **Rate** | **Number** | **Rate** | **Number** | **Rate** | **Number** | **Rate** | **Number** | **Rate** | **Number** | **Rate** |  |
| **Andean Latin America** | **Both** | 2021 (1796 to 2243) | 10 (8.8 to 11.1) | 1523 (1345 to 1690) | 7.9 (7 to 8.8) | 38034 (33889 to 42370) | 173.8 (154.3 to 193.6) | 11094 (8935 to 13467) | 20 (16.1 to 24.2) | 5630 (4593 to 6791) | 10.3 (8.4 to 12.4) | 125578 (101753 to 151796) | 220.8 (179 to 266.3) |  |
|  | **Female** | 1044 (930 to 1163) | 10.1 (9 to 11.2) | 827 (737 to 918) | 8.4 (7.5 to 9.3) | 19952 (17729 to 22276) | 179.3 (159.6 to 200) | 5570 (4500 to 6712) | 19.3 (15.6 to 23.2) | 3064 (2493 to 3655) | 10.7 (8.7 to 12.8) | 65223 (52423 to 78359) | 221.7 (178.4 to 266) |  |
|  | **Male** | 978 (843 to 1108) | 9.8 (8.5 to 11.1) | 696 (601 to 785) | 7.3 (6.3 to 8.3) | 18083 (15632 to 20422) | 167.8 (144.4 to 189.7) | 5524 (4311 to 6952) | 20.6 (16.1 to 25.9) | 2566 (2063 to 3181) | 9.9 (7.9 to 12.2) | 60355 (48005 to 75392) | 219.1 (174.6 to 273.9) |  |
| **Australasia** | **Both** | 12029 (11521 to 12422) | 51.6 (49.3 to 53.2) | 5639 (5377 to 5814) | 24.4 (23.2 to 25.2) | 126151 (121965 to 129715) | 546.9 (528.4 to 562.2) | 23671 (19439 to 28848) | 48.3 (39.6 to 59.1) | 8382 (7575 to 8978) | 16.2 (14.8 to 17.3) | 163248 (150872 to 173959) | 348.6 (324.2 to 370.7) |  |
|  | **Female** | 5775 (5441 to 6054) | 44.9 (42.4 to 47) | 2733 (2546 to 2855) | 20.9 (19.5 to 21.8) | 57987 (55376 to 60069) | 468.2 (448.7 to 484.8) | 10772 (8740 to 13129) | 41.1 (33.3 to 50.3) | 3931 (3426 to 4322) | 13.7 (12.2 to 14.9) | 72422 (65192 to 78284) | 292.2 (266 to 314.7) |  |
|  | **Male** | 6255 (5996 to 6496) | 59.9 (57.3 to 62.3) | 2906 (2797 to 2998) | 29 (27.8 to 30) | 68164 (65844 to 70404) | 640.6 (618.8 to 661.4) | 12899 (10469 to 15961) | 56.4 (45.7 to 69.7) | 4450 (4088 to 4788) | 19.1 (17.6 to 20.5) | 90826 (84077 to 97797) | 410.4 (381.2 to 441.7) |  |
| **Caribbean** | **Both** | 4690 (4486 to 4854) | 18.2 (17.3 to 18.8) | 3286 (3113 to 3415) | 13.1 (12.4 to 13.6) | 75932 (72040 to 78983) | 285.8 (271.2 to 297.3) | 13813 (11813 to 15959) | 26.7 (22.9 to 30.9) | 7995 (6935 to 9176) | 15.5 (13.4 to 17.7) | 172016 (147186 to 200175) | 333.3 (285.2 to 387.9) |  |
|  | **Female** | 2473 (2327 to 2596) | 18.5 (17.4 to 19.4) | 1732 (1615 to 1829) | 13.3 (12.3 to 14) | 39062 (36493 to 41494) | 285 (266.2 to 302.5) | 7154 (6160 to 8232) | 25.8 (22.2 to 29.7) | 4151 (3565 to 4759) | 14.8 (12.7 to 16.9) | 84882 (72294 to 98464) | 310.5 (264.5 to 361) |  |
|  | **Male** | 2218 (2116 to 2317) | 17.8 (17 to 18.7) | 1554 (1476 to 1629) | 12.9 (12.2 to 13.5) | 36870 (35136 to 38826) | 286.3 (272.7 to 301.5) | 6658 (5622 to 7775) | 27.6 (23.4 to 32.3) | 3844 (3278 to 4484) | 16.2 (13.7 to 18.8) | 87134 (72823 to 102378) | 357.6 (299.5 to 419.7) |  |
| **Central Asia** | **Both** | 6746 (6520 to 6979) | 14 (13.5 to 14.5) | 5122 (4952 to 5301) | 11 (10.6 to 11.3) | 147813 (143120 to 153238) | 292.8 (283.4 to 303.2) | 10949 (9999 to 12008) | 15.2 (13.9 to 16.6) | 7467 (6822 to 8166) | 11.2 (10.3 to 12.2) | 199841 (182012 to 219941) | 256.8 (234.4 to 281.1) |  |
|  | **Female** | 3443 (3295 to 3605) | 12.3 (11.8 to 12.9) | 2656 (2540 to 2780) | 9.6 (9.2 to 10.1) | 71800 (68789 to 75234) | 253.6 (242.9 to 265.8) | 5343 (4841 to 5898) | 13.2 (12 to 14.6) | 3699 (3364 to 4086) | 9.7 (8.8 to 10.7) | 93541 (84539 to 103665) | 218.8 (198.5 to 241.4) |  |
|  | **Male** | 3303 (3196 to 3422) | 16.4 (15.8 to 17) | 2466 (2387 to 2556) | 13 (12.5 to 13.4) | 76013 (73635 to 78775) | 345.6 (334.6 to 358.1) | 5606 (5097 to 6192) | 18 (16.4 to 19.7) | 3768 (3431 to 4161) | 13.3 (12.1 to 14.6) | 106300 (96592 to 117728) | 306 (279.3 to 337.1) |  |
| **Central Europe** | **Both** | 41586 (40372 to 42590) | 28.4 (27.5 to 29.1) | 30827 (29768 to 31602) | 21.5 (20.7 to 22.1) | 716519 (699250 to 732012) | 486.3 (474.1 to 497.2) | 84474 (74551 to 95453) | 39.9 (35.2 to 45.1) | 51567 (45636 to 57749) | 23.6 (20.8 to 26.4) | 1052146 (922923 to 1184246) | 512.6 (448.7 to 577.9) |  |
|  | **Female** | 19422 (18704 to 20048) | 23.3 (22.4 to 24) | 14787 (14101 to 15285) | 17.8 (16.9 to 18.4) | 325707 (314518 to 335795) | 395.6 (381.9 to 408) | 34732 (30180 to 39428) | 28.9 (25.1 to 32.9) | 22323 (19509 to 25128) | 17.2 (15 to 19.4) | 422629 (367973 to 480530) | 368.3 (320.2 to 420.8) |  |
|  | **Male** | 22164 (21607 to 22706) | 35.4 (34.4 to 36.3) | 16040 (15622 to 16425) | 26.8 (26 to 27.5) | 390811 (381891 to 400052) | 604.3 (590.1 to 619.3) | 49742 (43242 to 56900) | 54.4 (47.4 to 62.1) | 29244 (25557 to 33079) | 32.4 (28.4 to 36.6) | 629517 (543461 to 714960) | 694.4 (601.1 to 789.8) |  |
| **Central Latin America** | **Both** | 7477 (7204 to 7656) | 9 (8.6 to 9.3) | 5732 (5482 to 5890) | 7.3 (6.9 to 7.5) | 148160 (144013 to 151426) | 162.3 (156.8 to 166.1) | 37542 (32211 to 43870) | 15.9 (13.7 to 18.6) | 22470 (19542 to 25997) | 9.7 (8.4 to 11.2) | 539638 (465200 to 627069) | 223.7 (193.1 to 259.5) |  |
|  | **Female** | 4024 (3855 to 4148) | 9.4 (8.9 to 9.7) | 3101 (2938 to 3205) | 7.7 (7.2 to 8) | 77484 (75056 to 79656) | 166 (159.7 to 171) | 18489 (15530 to 22074) | 14.6 (12.3 to 17.4) | 11232 (9608 to 13238) | 8.9 (7.7 to 10.5) | 257899 (220325 to 305862) | 200.2 (171 to 237.2) |  |
|  | **Male** | 3452 (3319 to 3541) | 8.5 (8.1 to 8.8) | 2631 (2526 to 2703) | 6.9 (6.5 to 7.1) | 70677 (68544 to 72312) | 158.1 (152.8 to 162.2) | 19053 (16147 to 22441) | 17.5 (14.8 to 20.6) | 11238 (9576 to 13218) | 10.6 (9 to 12.4) | 281739 (238586 to 332736) | 250.2 (212.2 to 295.1) |  |
| **Central Sub-Saharan Africa** | **Both** | 1612 (1255 to 2045) | 7.4 (5.9 to 9.3) | 1515 (1209 to 1898) | 7.5 (6 to 9.4) | 43590 (34563 to 54900) | 173.8 (138.8 to 217.3) | 3957 (3015 to 5113) | 7.7 (5.9 to 10.1) | 3544 (2705 to 4609) | 7.4 (5.7 to 9.9) | 100988 (75749 to 131447) | 169.3 (129.2 to 220.2) |  |
|  | **Female** | 730 (548 to 964) | 6.4 (4.9 to 8.2) | 678 (521 to 884) | 6.4 (5 to 8.2) | 19648 (14666 to 26138) | 147.7 (113.2 to 192) | 1874 (1414 to 2546) | 6.7 (5 to 9.1) | 1693 (1274 to 2289) | 6.4 (4.8 to 9) | 46582 (34918 to 62552) | 146 (109.6 to 197.7) |  |
|  | **Male** | 883 (660 to 1243) | 8.6 (6.5 to 12.6) | 837 (639 to 1163) | 8.8 (6.7 to 13.2) | 23942 (18103 to 33085) | 203.4 (155.2 to 286.8) | 2082 (1575 to 3160) | 9.1 (6.9 to 14.2) | 1851 (1404 to 2828) | 8.8 (6.7 to 14.1) | 54407 (40546 to 81967) | 198.8 (151.2 to 306.1) |  |
| **East Asia** | **Both** | 112326 (100313 to 125624) | 12.8 (11.4 to 14.3) | 83307 (73752 to 93316) | 10.3 (9.2 to 11.4) | 2385455 (2099377 to 2681229) | 248.6 (219.6 to 278.4) | 637096 (548895 to 738549) | 30.9 (26.8 to 35.7) | 275604 (238238 to 317886) | 14.1 (12.2 to 16.2) | 6712862 (5774277 to 7735907) | 325.2 (280.7 to 373.2) |  |
|  | **Female** | 51901 (43537 to 60252) | 11.5 (9.7 to 13.3) | 39121 (33065 to 45365) | 9.2 (7.8 to 10.6) | 1083685 (910091 to 1262893) | 225 (190.2 to 261.4) | 229472 (186977 to 279731) | 21.4 (17.5 to 26.1) | 102931 (84137 to 122899) | 9.9 (8.1 to 11.8) | 2350983 (1928623 to 2824453) | 221.2 (181.5 to 265.8) |  |
|  | **Male** | 60425 (51025 to 70241) | 14.5 (12.4 to 16.7) | 44186 (37140 to 51745) | 11.9 (10.2 to 13.7) | 1301770 (1087443 to 1536741) | 276.8 (234.1 to 323.4) | 407624 (327106 to 501191) | 41.9 (33.9 to 51) | 172673 (140446 to 210292) | 19.6 (16.2 to 23.3) | 4361879 (3530820 to 5332752) | 440 (361.5 to 533.7) |  |
| **Eastern Europe** | **Both** | 70401 (68287 to 72718) | 25.1 (24.3 to 25.9) | 49828 (48379 to 51349) | 18 (17.4 to 18.6) | 1231358 (1191177 to 1269242) | 438.4 (423.8 to 452.1) | 106017 (96250 to 117074) | 31.1 (28.2 to 34.4) | 63476 (57180 to 70011) | 18.3 (16.5 to 20.2) | 1419105 (1287540 to 1571374) | 423.7 (384 to 469.3) |  |
|  | **Female** | 41094 (39711 to 43119) | 23 (22.2 to 24.2) | 28685 (27678 to 29996) | 15.8 (15.2 to 16.5) | 671246 (648965 to 704317) | 385.7 (372.8 to 405.2) | 56911 (49618 to 65078) | 27.2 (23.6 to 31.2) | 33518 (29181 to 37897) | 15.1 (13.1 to 17.1) | 701456 (613335 to 799604) | 346.5 (302.1 to 397.2) |  |
|  | **Male** | 29307 (28212 to 30280) | 29.7 (28.7 to 30.7) | 21143 (20436 to 21797) | 23 (22.3 to 23.7) | 560112 (538335 to 579137) | 538.9 (518.9 to 557.2) | 49106 (42532 to 55954) | 37.7 (32.8 to 42.8) | 29957 (26178 to 34025) | 24 (21.1 to 27.2) | 717649 (626209 to 819145) | 544.8 (476.5 to 620.1) |  |
| **Eastern Sub-Saharan Africa** | **Both** | 5196 (4336 to 6144) | 7 (5.8 to 8.2) | 4900 (4103 to 5787) | 7 (5.9 to 8.2) | 140164 (116319 to 167138) | 167.2 (139.9 to 198.3) | 14227 (12130 to 16886) | 8.8 (7.6 to 10.4) | 12717 (10940 to 15001) | 8.5 (7.4 to 9.9) | 356433 (301931 to 425606) | 193.9 (166 to 229.6) |  |
|  | **Female** | 2411 (1876 to 3087) | 6.4 (4.9 to 8) | 2245 (1736 to 2863) | 6.3 (4.9 to 8) | 65772 (49773 to 85467) | 152 (116.8 to 195) | 6764 (5643 to 7998) | 8 (6.7 to 9.4) | 6087 (5120 to 7162) | 7.7 (6.5 to 9) | 168860 (139126 to 201347) | 175.7 (146.9 to 208) |  |
|  | **Male** | 2785 (2253 to 3680) | 7.7 (6.3 to 9.8) | 2655 (2151 to 3499) | 7.8 (6.4 to 9.9) | 74392 (60289 to 99916) | 182.6 (148 to 240.9) | 7463 (6333 to 9390) | 9.8 (8.4 to 12.1) | 6630 (5656 to 8225) | 9.4 (8.1 to 11.6) | 187574 (157641 to 236113) | 213.9 (181.6 to 266.2) |  |
| **High-income Asia Pacific** | **Both** | 77180 (73961 to 79223) | 38.7 (36.9 to 39.8) | 34338 (32599 to 35180) | 17.9 (16.9 to 18.4) | 806452 (779644 to 825250) | 398.3 (383.4 to 408.3) | 196371 (166417 to 225643) | 44.6 (38.4 to 51.1) | 76929 (64821 to 83603) | 15.3 (13.4 to 16.4) | 1327823 (1186117 to 1414814) | 323.9 (298.6 to 342.1) |  |
|  | **Female** | 33579 (31575 to 34792) | 29.8 (27.9 to 30.9) | 16057 (14905 to 16620) | 14.5 (13.4 to 15) | 355782 (338782 to 366391) | 319 (303.5 to 328.5) | 81557 (64525 to 96207) | 32.3 (26.5 to 38) | 36551 (28651 to 41177) | 11.7 (9.7 to 12.8) | 553739 (466489 to 604656) | 241.8 (217.1 to 258) |  |
|  | **Male** | 43601 (42231 to 44805) | 50.8 (48.9 to 52.2) | 18281 (17682 to 18615) | 22.9 (22 to 23.5) | 450670 (439789 to 460181) | 501.5 (487 to 512.8) | 114815 (96227 to 136829) | 58.7 (49.6 to 70) | 40379 (36407 to 42849) | 19.6 (17.8 to 20.7) | 774085 (718825 to 817126) | 416.2 (389.6 to 438.8) |  |
| **High-income North America** | **Both** | 167902 (160795 to 172260) | 47.5 (45.6 to 48.6) | 71908 (67815 to 74170) | 20 (18.9 to 20.6) | 1508389 (1456315 to 1550409) | 440.6 (426.2 to 452.4) | 260911 (229909 to 295693) | 42.7 (37.6 to 48.6) | 95664 (88321 to 99688) | 14.9 (13.9 to 15.5) | 1987109 (1895869 to 2059774) | 339.9 (325.9 to 351.9) |  |
|  | **Female** | 82721 (77795 to 85772) | 40.2 (38.2 to 41.5) | 36692 (33894 to 38244) | 17 (15.9 to 17.7) | 719795 (685395 to 744260) | 370.9 (356.4 to 382.1) | 119679 (100934 to 141909) | 35.8 (30.2 to 42.8) | 45726 (40902 to 48394) | 12.6 (11.5 to 13.2) | 880514 (824050 to 922108) | 280.4 (265.5 to 292.4) |  |
|  | **Male** | 85181 (82614 to 87136) | 57.2 (55.4 to 58.6) | 35216 (33929 to 36142) | 24.2 (23.2 to 24.9) | 788593 (764985 to 809754) | 529.8 (513.4 to 544) | 141232 (118610 to 168934) | 50.5 (42.4 to 60.4) | 49938 (47441 to 51674) | 17.7 (16.8 to 18.4) | 1106595 (1063058 to 1147353) | 406.4 (390.8 to 420.7) |  |
| **North Africa and Middle East** | **Both** | 15426 (12968 to 18178) | 9 (7.6 to 10.6) | 13079 (11006 to 15347) | 8.2 (6.9 to 9.6) | 365144 (308288 to 432794) | 192.8 (162.7 to 227) | 60010 (53354 to 67555) | 13.9 (12.3 to 15.6) | 39147 (34761 to 44107) | 9.8 (8.7 to 11) | 1013634 (896161 to 1146526) | 218.7 (194.1 to 246.5) |  |
|  | **Female** | 7001 (5969 to 8367) | 8.3 (7.1 to 9.9) | 6284 (5345 to 7497) | 7.9 (6.7 to 9.4) | 173195 (147430 to 209960) | 185.9 (158.4 to 223.3) | 23539 (20661 to 26773) | 11.4 (10 to 13) | 17851 (15651 to 20307) | 9.2 (8 to 10.4) | 451809 (394611 to 519371) | 200.4 (175.5 to 229.5) |  |
|  | **Male** | 8425 (6817 to 10337) | 9.6 (7.8 to 11.8) | 6795 (5504 to 8311) | 8.4 (6.8 to 10.2) | 191949 (156002 to 236808) | 199.3 (161.6 to 244.3) | 36471 (31820 to 41687) | 16.3 (14.3 to 18.6) | 21296 (18681 to 24218) | 10.5 (9.2 to 11.9) | 561825 (488138 to 644361) | 236.2 (206.2 to 269.5) |  |
| **Oceania** | **Both** | 246 (194 to 295) | 8.3 (6.6 to 9.9) | 206 (161 to 248) | 7.7 (6.1 to 9.2) | 6204 (4858 to 7521) | 182.2 (142.6 to 218.4) | 691 (555 to 855) | 10 (8.2 to 12.1) | 551 (443 to 682) | 8.8 (7.2 to 10.7) | 16315 (12915 to 20556) | 203.6 (163.6 to 252.5) |  |
|  | **Female** | 110 (85 to 136) | 7.8 (6 to 9.7) | 93 (71 to 116) | 7.3 (5.6 to 9.2) | 2727 (2077 to 3401) | 168.5 (128.6 to 209.7) | 307 (244 to 389) | 9.3 (7.5 to 11.5) | 248 (197 to 312) | 8.2 (6.6 to 10.1) | 7105 (5544 to 9194) | 186.2 (148.3 to 235.5) |  |
|  | **Male** | 136 (105 to 171) | 8.9 (6.9 to 11) | 113 (86 to 142) | 8.2 (6.4 to 10.3) | 3476 (2665 to 4422) | 195.3 (150.1 to 245.1) | 384 (304 to 480) | 10.7 (8.6 to 13.1) | 303 (238 to 378) | 9.5 (7.7 to 11.7) | 9210 (7131 to 11787) | 220.3 (173.8 to 275.3) |  |
| **South Asia** | **Both** | 29941 (26436 to 34063) | 5.4 (4.8 to 6.2) | 27309 (24144 to 31138) | 5.3 (4.7 to 6.1) | 786708 (697212 to 893701) | 125.4 (111.1 to 142.9) | 113711 (98190 to 129352) | 8.3 (7.2 to 9.4) | 94846 (81524 to 109075) | 7.3 (6.2 to 8.3) | 2419098 (2078019 to 2782570) | 165.1 (141.7 to 189.9) |  |
|  | **Female** | 14293 (11595 to 17221) | 5.4 (4.3 to 6.5) | 12934 (10399 to 15765) | 5.2 (4.1 to 6.4) | 381425 (311417 to 463542) | 125.6 (101.4 to 153.2) | 57972 (47453 to 68859) | 8.3 (6.8 to 9.9) | 48508 (39745 to 57826) | 7.3 (6 to 8.6) | 1232829 (1008947 to 1474885) | 166.2 (136.3 to 199.3) |  |
|  | **Male** | 15649 (13407 to 19374) | 5.5 (4.7 to 6.8) | 14374 (12380 to 17850) | 5.4 (4.6 to 6.7) | 405284 (351998 to 503879) | 125.2 (108 to 155) | 55739 (45824 to 65822) | 8.3 (6.9 to 9.8) | 46338 (38277 to 54856) | 7.3 (6.1 to 8.6) | 1186269 (975244 to 1412736) | 164 (135.6 to 194.2) |  |
| **Southeast Asia** | **Both** | 27898 (24572 to 30662) | 10.8 (9.6 to 11.8) | 23639 (20959 to 25972) | 9.8 (8.7 to 10.7) | 672882 (590895 to 746284) | 235.9 (208.4 to 260.1) | 117010 (96631 to 136244) | 19.3 (16 to 22.4) | 82024 (67617 to 94606) | 14.4 (11.9 to 16.6) | 2142434 (1780490 to 2482287) | 334 (276.6 to 386.4) |  |
|  | **Female** | 13634 (11483 to 15452) | 10 (8.5 to 11.2) | 11641 (9853 to 13248) | 9.1 (7.8 to 10.2) | 324446 (269196 to 374534) | 216.5 (182.4 to 248) | 51266 (40497 to 61454) | 15.9 (12.6 to 18.9) | 36725 (28821 to 44227) | 11.9 (9.4 to 14.3) | 910528 (704666 to 1108872) | 269.9 (209.9 to 327.4) |  |
|  | **Male** | 14264 (12731 to 16061) | 11.8 (10.5 to 13.2) | 11998 (10814 to 13651) | 10.7 (9.7 to 12.1) | 348436 (311833 to 397472) | 257.8 (231.9 to 293.3) | 65744 (54302 to 76879) | 23.3 (19.3 to 27.3) | 45299 (37265 to 52887) | 17.4 (14.2 to 20.3) | 1231906 (1009547 to 1446139) | 406.9 (334.9 to 475.8) |  |
| **Southern Latin America** | **Both** | 10929 (10519 to 11262) | 24.1 (23.1 to 24.8) | 8829 (8469 to 9093) | 20 (19 to 20.6) | 195300 (189163 to 200639) | 422.1 (407.7 to 433.7) | 26866 (21480 to 33612) | 32.2 (25.7 to 40.4) | 17930 (16774 to 18975) | 21.2 (19.9 to 22.4) | 366436 (347729 to 385441) | 447.6 (424.7 to 470.5) |  |
|  | **Female** | 5164 (4910 to 5383) | 20.3 (19.3 to 21.2) | 4260 (4017 to 4447) | 17 (16 to 17.8) | 89226 (85589 to 92507) | 351 (336.6 to 364) | 12551 (10139 to 15570) | 26.5 (21.3 to 33.1) | 8662 (7908 to 9340) | 17.5 (16.2 to 18.8) | 164879 (153863 to 175874) | 363 (338.6 to 387.8) |  |
|  | **Male** | 5765 (5540 to 5994) | 28.8 (27.6 to 30) | 4569 (4388 to 4741) | 23.7 (22.7 to 24.6) | 106074 (102566 to 109937) | 508.5 (491.1 to 526.5) | 14315 (11376 to 18014) | 39.4 (31.3 to 49.5) | 9269 (8700 to 9877) | 26 (24.3 to 27.7) | 201557 (189829 to 213993) | 550.2 (518.5 to 583.6) |  |
| **Southern Sub-Saharan Africa** | **Both** | 2868 (2504 to 3344) | 10.7 (9.3 to 12.7) | 2569 (2213 to 3044) | 10.2 (8.7 to 12.2) | 65678 (58004 to 75105) | 222.5 (193.9 to 259.8) | 7106 (6389 to 7882) | 13.1 (11.8 to 14.5) | 5922 (5329 to 6580) | 11.5 (10.4 to 12.7) | 147780 (132439 to 165539) | 250.4 (225.1 to 279.3) |  |
|  | **Female** | 1446 (1227 to 1695) | 9.6 (8 to 11.4) | 1330 (1116 to 1588) | 9.2 (7.6 to 11.2) | 31491 (27258 to 35744) | 193.2 (165.3 to 223.9) | 3469 (2993 to 3931) | 11 (9.5 to 12.5) | 2978 (2582 to 3379) | 9.8 (8.5 to 11.1) | 68699 (58762 to 78830) | 206.7 (177.8 to 236.7) |  |
|  | **Male** | 1423 (1224 to 1741) | 12.1 (10.4 to 15.1) | 1239 (1061 to 1545) | 11.3 (9.6 to 14.3) | 34187 (29483 to 41363) | 257.5 (220.9 to 319) | 3637 (3241 to 4118) | 16 (14.3 to 18) | 2944 (2627 to 3337) | 14 (12.6 to 15.7) | 79081 (70204 to 89934) | 308.8 (275.7 to 351.3) |  |
| **Tropical Latin America** | **Both** | 10717 (10338 to 11048) | 12 (11.5 to 12.4) | 8475 (8117 to 8749) | 10.1 (9.6 to 10.5) | 222096 (215287 to 228252) | 226.7 (218.3 to 233.6) | 42891 (40118 to 44928) | 17.8 (16.6 to 18.6) | 27704 (25668 to 29090) | 11.7 (10.8 to 12.3) | 660129 (625562 to 687740) | 268.3 (253.7 to 279.8) |  |
|  | **Female** | 5610 (5348 to 5840) | 11.8 (11.2 to 12.3) | 4472 (4235 to 4656) | 10 (9.3 to 10.4) | 113974 (109624 to 118148) | 221.4 (211.6 to 229.8) | 21231 (19519 to 22654) | 16 (14.7 to 17.1) | 13953 (12681 to 14886) | 10.5 (9.6 to 11.2) | 320149 (296982 to 338928) | 240.5 (223.1 to 254.5) |  |
|  | **Male** | 5107 (4932 to 5278) | 12.2 (11.7 to 12.6) | 4003 (3838 to 4150) | 10.2 (9.7 to 10.6) | 108122 (104281 to 111915) | 232.3 (223.1 to 240.3) | 21659 (20145 to 22968) | 19.9 (18.5 to 21.1) | 13751 (12825 to 14566) | 13.1 (12.1 to 13.9) | 339979 (319588 to 358604) | 301.4 (282.9 to 318) |  |
| **Western Europe** | **Both** | 229473 (220382 to 234886) | 39.6 (38 to 40.5) | 130906 (124426 to 134404) | 22.2 (21.1 to 22.8) | 2593189 (2510115 to 2651075) | 461.9 (448.2 to 471.8) | 382442 (332800 to 432448) | 42.4 (37.1 to 48.3) | 172454 (155345 to 181815) | 17.3 (15.8 to 18.1) | 3008234 (2815060 to 3152895) | 351.2 (332 to 366.8) |  |
|  | **Female** | 115530 (109076 to 119311) | 33.6 (32 to 34.6) | 68085 (63298 to 70657) | 18.8 (17.6 to 19.5) | 1251384 (1191566 to 1287972) | 386.2 (370.7 to 396.6) | 170406 (145276 to 194506) | 33.9 (29.2 to 38.9) | 81128 (70782 to 87193) | 13.8 (12.4 to 14.6) | 1302984 (1189657 to 1382605) | 276.1 (257.7 to 290.7) |  |
|  | **Male** | 113943 (110951 to 116371) | 48.1 (46.7 to 49.1) | 62821 (60966 to 64175) | 27.4 (26.4 to 28) | 1341804 (1311121 to 1368394) | 563.8 (549.9 to 575.1) | 212035 (185239 to 240969) | 52.6 (45.8 to 59.9) | 91326 (85302 to 95966) | 21.8 (20.4 to 22.8) | 1705250 (1615826 to 1783738) | 438.3 (416.6 to 458.2) |  |
| **Western Sub-Saharan Africa** | **Both** | 5434 (4402 to 6640) | 6.5 (5.3 to 8) | 5189 (4250 to 6327) | 6.6 (5.4 to 8) | 132928 (106960 to 162799) | 143.8 (117.1 to 175.6) | 15321 (12895 to 17824) | 8.7 (7.4 to 10) | 13773 (11698 to 16069) | 8.4 (7.3 to 9.7) | 353242 (295571 to 420704) | 176.1 (149 to 206.2) |  |
|  | **Female** | 2490 (1931 to 3217) | 6.1 (4.7 to 7.8) | 2403 (1875 to 3066) | 6.1 (4.8 to 7.8) | 58752 (45821 to 76529) | 130.8 (101.8 to 169.4) | 7373 (6002 to 8817) | 8.2 (6.8 to 9.6) | 6662 (5494 to 7954) | 7.9 (6.6 to 9.3) | 166786 (134494 to 203272) | 161.5 (132.4 to 193.6) |  |
|  | **Male** | 2944 (2310 to 3735) | 7 (5.6 to 8.7) | 2787 (2200 to 3495) | 7.1 (5.6 to 8.7) | 74176 (57957 to 94509) | 156 (122.8 to 196.2) | 7948 (6543 to 9543) | 9.3 (7.8 to 11.1) | 7111 (5811 to 8652) | 8.9 (7.4 to 10.7) | 186456 (150166 to 230347) | 192 (156.2 to 235.4) |  |

Data in parentheses are 95% uncertainty intervals

Abbreviations: SDI = socio-demographic index, DALYs = disability-adjusted life years
