# Supplementary material for: The global, regional, and national burden and quality of care index (QCI) of colorectal cancer; a global burden of disease systematic analysis 1990–2019
Source: PLoS One. 2022 Apr 21;17(4):e0263403. doi: 10.1371/journal.pone.0263403 (PMC9022854; doi:10.1371/journal.pone.0263403)
Supplement: S4 Table — (DOCX) [file pone.0263403.s004.docx]

**S4 Table.** Quality of care index (QCI) in the world, World Health Organization Regions, sociodemographic index (SDI) quintiles, and countries in both sexes in 1990 and 2019.

| **Location type** | **Location name** | **QCI** | |
| --- | --- | --- | --- |
|  |  | **1990** | **2019** |
| **Global** | **Global** | 63.6 | 77.6 |
| **WHO Regions** | **African Region** | 13.0 | 23.6 |
|  | **Eastern Mediterranean Region** | 23.2 | 45.5 |
|  | **European Region** | 64.8 | 81.3 |
|  | **Region of the Americas** | 79.5 | 84.4 |
|  | **South-East Asia Region** | 18.7 | 36.5 |
|  | **Western Pacific Region** | 61.5 | 84.3 |
| **SDI** | **High SDI** | 79.8 | 57.6 |
|  | **High-middle SDI** | 54.8 | 53.5 |
|  | **Middle SDI** | 34.9 | 49.0 |
|  | **Low-middle SDI** | 19.2 | 41.4 |
|  | **Low SDI** | 8.2 | 24.9 |
| **Country** | **Afghanistan** | 4.3 | 10.9 |
|  | **Albania** | 42.1 | 67.6 |
|  | **Algeria** | 29.5 | 50.7 |
|  | **American Samoa** | 31.8 | 41.9 |
|  | **Andorra** | 76.1 | 90.9 |
|  | **Angola** | 3.7 | 15.8 |
|  | **Antigua and Barbuda** | 48.4 | 63.3 |
|  | **Argentina** | 39.4 | 58.3 |
|  | **Armenia** | 41.8 | 58.6 |
|  | **Australia** | 82.4 | 97.8 |
|  | **Austria** | 74.6 | 90.8 |
|  | **Azerbaijan** | 34.0 | 46.2 |
|  | **Bahamas** | 45.1 | 57.3 |
|  | **Bahrain** | 31.7 | 61.4 |
|  | **Bangladesh** | 8.4 | 31.0 |
|  | **Barbados** | 49.7 | 66.1 |
|  | **Belarus** | 56.8 | 76.8 |
|  | **Belgium** | 69.3 | 87.8 |
|  | **Belize** | 38.0 | 51.1 |
|  | **Benin** | 9.3 | 16.0 |
|  | **Bermuda** | 58.8 | 85.1 |
|  | **Bhutan** | 7.9 | 33.0 |
|  | **Bolivia (Plurinational State of)** | 23.1 | 50.3 |
|  | **Bosnia and Herzegovina** | 42.6 | 62.1 |
|  | **Botswana** | 18.2 | 35.2 |
|  | **Brazil** | 34.0 | 58.0 |
|  | **Brunei Darussalam** | 50.5 | 67.1 |
|  | **Bulgaria** | 54.3 | 68.3 |
|  | **Burkina Faso** | 8.5 | 15.5 |
|  | **Burundi** | 3.3 | 12.9 |
|  | **Cabo Verde** | 24.1 | 39.1 |
|  | **Cambodia** | 12.2 | 33.3 |
|  | **Cameroon** | 9.2 | 18.8 |
|  | **Canada** | 86.7 | 97.5 |
|  | **Central African Republic** | 1.7 | 4.6 |
|  | **Chad** | 7.8 | 10.4 |
|  | **Chile** | 44.2 | 70.8 |
|  | **China** | 41.9 | 82.6 |
|  | **Colombia** | 40.1 | 70.0 |
|  | **Comoros** | 7.0 | 18.0 |
|  | **Congo** | 5.5 | 16.7 |
|  | **Cook Islands** | 47.0 | 64.1 |
|  | **Costa Rica** | 58.5 | 73.8 |
|  | **Côte d'Ivoire** | 9.2 | 15.3 |
|  | **Croatia** | 60.7 | 78.0 |
|  | **Cuba** | 58.2 | 75.7 |
|  | **Cyprus** | 68.2 | 93.2 |
|  | **Czechia** | 53.8 | 77.9 |
|  | **Democratic People's Republic of Korea** | 44.5 | 51.1 |
|  | **Democratic Republic of the Congo** | 7.1 | 14.2 |
|  | **Denmark** | 65.4 | 85.8 |
|  | **Djibouti** | 10.6 | 20.6 |
|  | **Dominica** | 43.1 | 47.4 |
|  | **Dominican Republic** | 29.4 | 49.8 |
|  | **Ecuador** | 38.4 | 67.8 |
|  | **Egypt** | 22.7 | 43.8 |
|  | **El Salvador** | 32.0 | 59.0 |
|  | **Equatorial Guinea** | 2.9 | 24.8 |
|  | **Eritrea** | 0.9 | 12.0 |
|  | **Estonia** | 62.3 | 85.2 |
|  | **Eswatini** | 13.4 | 18.4 |
|  | **Ethiopia** | 3.5 | 19.3 |
|  | **Fiji** | 27.2 | 33.0 |
|  | **Finland** | 73.8 | 91.5 |
|  | **France** | 69.2 | 89.1 |
|  | **Gabon** | 11.7 | 25.0 |
|  | **Gambia** | 12.8 | 18.0 |
|  | **Georgia** | 49.5 | 52.8 |
|  | **Germany** | 73.3 | 87.7 |
|  | **Ghana** | 13.3 | 23.0 |
|  | **Greece** | 75.9 | 86.2 |
|  | **Greenland** | 42.8 | 59.7 |
|  | **Grenada** | 36.8 | 54.1 |
|  | **Guam** | 45.8 | 56.8 |
|  | **Guatemala** | 22.1 | 42.0 |
|  | **Guinea** | 7.4 | 12.5 |
|  | **Guinea-Bissau** | 4.6 | 11.8 |
|  | **Guyana** | 27.0 | 39.5 |
|  | **Haiti** | 9.0 | 20.6 |
|  | **Honduras** | 25.0 | 41.3 |
|  | **Hungary** | 50.6 | 72.9 |
|  | **Iceland** | 81.4 | 92.7 |
|  | **India** | 13.5 | 29.9 |
|  | **Indonesia** | 21.5 | 35.4 |
|  | **Iran (Islamic Republic of)** | 36.0 | 59.4 |
|  | **Iraq** | 25.2 | 48.4 |
|  | **Ireland** | 69.6 | 91.7 |
|  | **Israel** | 61.6 | 82.6 |
|  | **Italy** | 80.2 | 95.5 |
|  | **Jamaica** | 47.7 | 60.1 |
|  | **Japan** | 86.8 | 98.2 |
|  | **Jordan** | 29.7 | 58.0 |
|  | **Kazakhstan** | 39.2 | 55.5 |
|  | **Kenya** | 17.1 | 18.3 |
|  | **Kiribati** | 11.7 | 17.8 |
|  | **Kuwait** | 54.0 | 73.2 |
|  | **Kyrgyzstan** | 34.4 | 45.9 |
|  | **Lao People's Democratic Republic** | 8.5 | 25.7 |
|  | **Latvia** | 52.3 | 69.7 |
|  | **Lebanon** | 33.1 | 68.4 |
|  | **Lesotho** | 12.9 | 12.1 |
|  | **Liberia** | 7.5 | 17.0 |
|  | **Libya** | 32.5 | 48.6 |
|  | **Lithuania** | 57.2 | 69.3 |
|  | **Luxembourg** | 69.0 | 89.5 |
|  | **Madagascar** | 8.5 | 14.4 |
|  | **Malawi** | 10.0 | 17.0 |
|  | **Malaysia** | 30.3 | 56.8 |
|  | **Maldives** | 25.0 | 66.3 |
|  | **Mali** | 7.2 | 16.3 |
|  | **Malta** | 69.5 | 87.5 |
|  | **Marshall Islands** | 17.5 | 24.4 |
|  | **Mauritania** | 9.5 | 24.0 |
|  | **Mauritius** | 43.5 | 60.8 |
|  | **Mexico** | 38.6 | 62.2 |
|  | **Micronesia (Federated States of)** | 18.4 | 32.1 |
|  | **Monaco** | 80.2 | 88.5 |
|  | **Mongolia** | 21.6 | 36.1 |
|  | **Montenegro** | 61.1 | 70.7 |
|  | **Morocco** | 20.6 | 38.6 |
|  | **Mozambique** | 5.5 | 12.1 |
|  | **Myanmar** | 12.3 | 31.6 |
|  | **Namibia** | 13.1 | 28.6 |
|  | **Nauru** | 30.4 | 41.8 |
|  | **Nepal** | 6.1 | 24.0 |
|  | **Netherlands** | 77.5 | 91.3 |
|  | **New Zealand** | 80.8 | 94.0 |
|  | **Nicaragua** | 34.9 | 59.5 |
|  | **Niger** | 6.3 | 13.8 |
|  | **Nigeria** | 13.8 | 21.6 |
|  | **Niue** | 38.3 | 56.2 |
|  | **North Macedonia** | 45.8 | 64.1 |
|  | **Northern Mariana Islands** | 50.6 | 63.6 |
|  | **Norway** | 74.9 | 91.6 |
|  | **Oman** | 36.7 | 61.9 |
|  | **Pakistan** | 13.0 | 22.8 |
|  | **Palau** | 42.0 | 55.7 |
|  | **Palestine** | 30.5 | 47.8 |
|  | **Panama** | 48.5 | 67.7 |
|  | **Papua New Guinea** | 14.0 | 19.9 |
|  | **Paraguay** | 32.3 | 52.6 |
|  | **Peru** | 49.1 | 83.7 |
|  | **Philippines** | 29.4 | 39.9 |
|  | **Poland** | 31.3 | 55.9 |
|  | **Portugal** | 59.8 | 86.9 |
|  | **Puerto Rico** | 61.2 | 83.5 |
|  | **Qatar** | 32.7 | 64.6 |
|  | **Republic of Korea** | 59.7 | 93.4 |
|  | **Republic of Moldova** | 45.9 | 63.5 |
|  | **Romania** | 47.5 | 69.8 |
|  | **Russian Federation** | 51.8 | 68.4 |
|  | **Rwanda** | 2.9 | 20.0 |
|  | **Saint Kitts and Nevis** | 46.3 | 64.0 |
|  | **Saint Lucia** | 37.9 | 57.5 |
|  | **Saint Vincent and the Grenadines** | 39.4 | 48.8 |
|  | **Samoa** | 31.0 | 42.9 |
|  | **San Marino** | 77.4 | 88.3 |
|  | **Sao Tome and Principe** | 12.7 | 24.4 |
|  | **Saudi Arabia** | 22.9 | 63.3 |
|  | **Senegal** | 9.4 | 17.1 |
|  | **Serbia** | 45.2 | 68.8 |
|  | **Seychelles** | 32.6 | 53.2 |
|  | **Sierra Leone** | 8.4 | 15.1 |
|  | **Singapore** | 71.1 | 94.5 |
|  | **Slovakia** | 61.7 | 81.1 |
|  | **Slovenia** | 60.6 | 81.9 |
|  | **Solomon Islands** | 20.3 | 30.1 |
|  | **Somalia** | 4.5 | 7.3 |
|  | **South Africa** | 23.1 | 33.3 |
|  | **South Sudan** | 5.4 | 9.6 |
|  | **Spain** | 76.5 | 93.1 |
|  | **Sri Lanka** | 36.0 | 64.9 |
|  | **Sudan** | 15.4 | 31.3 |
|  | **Suriname** | 31.2 | 44.8 |
|  | **Sweden** | 76.9 | 88.7 |
|  | **Switzerland** | 85.1 | 96.3 |
|  | **Syrian Arab Republic** | 30.8 | 52.2 |
|  | **Taiwan (Province of China)** | 63.9 | 86.7 |
|  | **Tajikistan** | 30.8 | 31.9 |
|  | **Thailand** | 34.0 | 66.0 |
|  | **Timor-Leste** | 13.6 | 28.6 |
|  | **Togo** | 12.3 | 18.4 |
|  | **Tokelau** | 25.3 | 44.7 |
|  | **Tonga** | 30.0 | 39.3 |
|  | **Trinidad and Tobago** | 38.3 | 57.1 |
|  | **Tunisia** | 33.9 | 59.3 |
|  | **Turkey** | 24.8 | 62.5 |
|  | **Turkmenistan** | 33.0 | 44.9 |
|  | **Tuvalu** | 20.3 | 34.3 |
|  | **Uganda** | 9.5 | 18.7 |
|  | **Ukraine** | 50.0 | 62.2 |
|  | **United Arab Emirates** | 27.1 | 44.4 |
|  | **United Kingdom** | 71.8 | 89.1 |
|  | **United Republic of Tanzania** | 11.1 | 20.0 |
|  | **United States of America** | 87.3 | 94.5 |
|  | **United States Virgin Islands** | 47.3 | 62.7 |
|  | **Uruguay** | 45.5 | 61.8 |
|  | **Uzbekistan** | 41.0 | 43.6 |
|  | **Vanuatu** | 17.8 | 22.2 |
|  | **Venezuela (Bolivarian Republic of)** | 40.4 | 63.4 |
|  | **Viet Nam** | 27.3 | 57.4 |
|  | **Yemen** | 15.4 | 26.0 |
|  | **Zambia** | 6.2 | 19.7 |
|  | **Zimbabwe** | 20.7 | 17.1 |

Abbreviations: QCI=quality of care index; SDI=socio-demographic index; WHO=Word Health Organization.
