# Supplementary material for: The global, regional, and national burden and quality of care index (QCI) of colorectal cancer; a global burden of disease systematic analysis 1990–2019
Source: PLoS One. 2022 Apr 21;17(4):e0263403. doi: 10.1371/journal.pone.0263403 (PMC9022854; doi:10.1371/journal.pone.0263403)
Supplement: S5 Table — (DOCX) [file pone.0263403.s005.docx]

**S5 Table.** Gender disparity ratio (GDR) values in the world, World Health Organization Regions, sociodemographic index (SDI) quintiles, and countries by sex in 1990 and 2019.

|  |  | **1990** | | | **2019** | | |
| --- | --- | --- | --- | --- | --- | --- | --- |
| **Location type** | **Location name** | **QCI** | | **GDR** | **QCI** | | **GDR** |
|  |  | **Females** | **Males** |  | **Females** | **Males** |  |
| **Global** | **Global** | 63.5 | 63.9 | 1.0 | 76.2 | 78.6 | 1.0 |
| **WHO Regions** | **African Region** | 13.3 | 14.4 | 0.9 | 22.8 | 25.7 | 0.9 |
|  | **Eastern Mediterranean Region** | 18.3 | 28.3 | 0.6 | 35.5 | 52.8 | 0.7 |
|  | **European Region** | 65.7 | 63.9 | 1.0 | 81.6 | 80.8 | 1.0 |
|  | **Region of the Americas** | 79.3 | 79.5 | 1.0 | 83.6 | 85.0 | 1.0 |
|  | **South-East Asia Region** | 18.9 | 19.9 | 1.0 | 36.1 | 37.7 | 1.0 |
|  | **Western Pacific Region** | 60.0 | 62.8 | 1.0 | 83.8 | 84.1 | 1.0 |
| **SDI** | **High SDI** | 79.8 | 79.4 | 1.0 | 57.3 | 58.1 | 1.0 |
|  | **High-middle SDI** | 56.0 | 53.8 | 1.0 | 53.0 | 54.1 | 1.0 |
|  | **Middle SDI** | 34.4 | 36.3 | 0.9 | 50.2 | 48.4 | 1.0 |
|  | **Low-middle SDI** | 19.4 | 20.5 | 0.9 | 39.9 | 43.6 | 0.9 |
|  | **Low SDI** | 8.4 | 9.8 | 0.9 | 25.8 | 25.2 | 1.0 |
| **Country** | **Afghanistan** | 0.3 | 10.1 | 0.0 | 5.3 | 18.6 | 0.3 |
|  | **Albania** | 44.1 | 40.9 | 1.1 | 67.8 | 67.4 | 1.0 |
|  | **Algeria** | 24.3 | 35.4 | 0.7 | 39.9 | 59.2 | 0.7 |
|  | **American Samoa** | 32.2 | 32.3 | 1.0 | 42.2 | 42.3 | 1.0 |
|  | **Andorra** | 78.5 | 74.9 | 1.0 | 91.7 | 89.9 | 1.0 |
|  | **Angola** | 5.1 | 4.5 | 1.1 | 16.8 | 16.3 | 1.0 |
|  | **Antigua and Barbuda** | 49.9 | 47.4 | 1.1 | 64.5 | 61.9 | 1.0 |
|  | **Argentina** | 40.3 | 39.5 | 1.0 | 58.7 | 58.1 | 1.0 |
|  | **Armenia** | 42.9 | 41.1 | 1.0 | 59.3 | 58.1 | 1.0 |
|  | **Australia** | 83.5 | 81.1 | 1.0 | 97.7 | 97.4 | 1.0 |
|  | **Austria** | 75.9 | 72.9 | 1.0 | 91.7 | 89.7 | 1.0 |
|  | **Azerbaijan** | 35.5 | 33.3 | 1.1 | 46.9 | 46.2 | 1.0 |
|  | **Bahamas** | 46.4 | 44.3 | 1.0 | 59.0 | 56.3 | 1.0 |
|  | **Bahrain** | 25.3 | 35.3 | 0.7 | 49.5 | 65.6 | 0.8 |
|  | **Bangladesh** | 8.4 | 10.2 | 0.8 | 30.9 | 32.2 | 1.0 |
|  | **Barbados** | 50.6 | 49.4 | 1.0 | 67.2 | 65.3 | 1.0 |
|  | **Belarus** | 58.7 | 54.3 | 1.1 | 79.0 | 73.7 | 1.1 |
|  | **Belgium** | 70.3 | 68.2 | 1.0 | 88.1 | 87.2 | 1.0 |
|  | **Belize** | 37.7 | 39.4 | 1.0 | 52.5 | 50.7 | 1.0 |
|  | **Benin** | 10.1 | 10.4 | 1.0 | 16.1 | 17.5 | 0.9 |
|  | **Bermuda** | 59.8 | 58.0 | 1.0 | 86.0 | 84.1 | 1.0 |
|  | **Bhutan** | 7.5 | 10.4 | 0.7 | 33.2 | 33.9 | 1.0 |
|  | **Bolivia (Plurinational State of)** | 22.0 | 26.0 | 0.8 | 49.3 | 52.1 | 0.9 |
|  | **Bosnia and Herzegovina** | 43.3 | 42.7 | 1.0 | 62.9 | 61.6 | 1.0 |
|  | **Botswana** | 18.8 | 19.5 | 1.0 | 34.8 | 36.6 | 0.9 |
|  | **Brazil** | 34.4 | 34.7 | 1.0 | 58.3 | 58.2 | 1.0 |
|  | **Brunei Darussalam** | 49.5 | 50.6 | 1.0 | 66.3 | 65.8 | 1.0 |
|  | **Bulgaria** | 56.1 | 53.2 | 1.1 | 70.0 | 67.0 | 1.0 |
|  | **Burkina Faso** | 8.5 | 10.3 | 0.8 | 15.5 | 17.1 | 0.9 |
|  | **Burundi** | 5.0 | 3.6 | 1.4 | 14.2 | 13.4 | 1.1 |
|  | **Cabo Verde** | 23.4 | 26.3 | 0.9 | 37.9 | 41.2 | 0.9 |
|  | **Cambodia** | 12.6 | 13.3 | 1.0 | 34.3 | 33.0 | 1.0 |
|  | **Cameroon** | 9.3 | 10.7 | 0.9 | 18.9 | 20.2 | 0.9 |
|  | **Canada** | 86.6 | 86.2 | 1.0 | 96.7 | 97.7 | 1.0 |
|  | **Central African Republic** | 4.5 | 1.8 | 2.5 | 6.3 | 5.2 | 1.2 |
|  | **Chad** | 8.0 | 9.5 | 0.8 | 10.3 | 12.2 | 0.8 |
|  | **Chile** | 43.7 | 45.7 | 1.0 | 70.5 | 71.2 | 1.0 |
|  | **China** | 41.8 | 42.4 | 1.0 | 82.7 | 81.6 | 1.0 |
|  | **Colombia** | 40.3 | 40.9 | 1.0 | 69.8 | 70.3 | 1.0 |
|  | **Comoros** | 8.1 | 7.6 | 1.1 | 18.9 | 18.2 | 1.0 |
|  | **Congo** | 6.9 | 6.0 | 1.1 | 17.0 | 18.0 | 0.9 |
|  | **Cook Islands** | 47.5 | 47.4 | 1.0 | 64.4 | 64.3 | 1.0 |
|  | **Costa Rica** | 58.8 | 58.5 | 1.0 | 73.6 | 74.1 | 1.0 |
|  | **Côte d'Ivoire** | 9.7 | 10.7 | 0.9 | 15.0 | 17.1 | 0.9 |
|  | **Croatia** | 63.7 | 57.7 | 1.1 | 81.3 | 75.0 | 1.1 |
|  | **Cuba** | 59.1 | 57.6 | 1.0 | 76.8 | 74.4 | 1.0 |
|  | **Cyprus** | 68.1 | 68.3 | 1.0 | 94.0 | 92.1 | 1.0 |
|  | **Czechia** | 55.6 | 52.4 | 1.1 | 78.5 | 77.2 | 1.0 |
|  | **Democratic People's Republic of Korea** | 45.2 | 43.5 | 1.0 | 52.1 | 50.0 | 1.0 |
|  | **Democratic Republic of the Congo** | 8.2 | 7.9 | 1.0 | 14.8 | 15.0 | 1.0 |
|  | **Denmark** | 66.2 | 64.9 | 1.0 | 85.6 | 85.6 | 1.0 |
|  | **Djibouti** | 11.4 | 11.4 | 1.0 | 21.7 | 21.2 | 1.0 |
|  | **Dominica** | 43.5 | 43.3 | 1.0 | 48.5 | 47.3 | 1.0 |
|  | **Dominican Republic** | 29.0 | 31.1 | 0.9 | 50.5 | 50.0 | 1.0 |
|  | **Ecuador** | 35.7 | 42.4 | 0.8 | 66.4 | 69.6 | 1.0 |
|  | **Egypt** | 18.5 | 27.9 | 0.7 | 31.2 | 52.8 | 0.6 |
|  | **El Salvador** | 32.2 | 33.0 | 1.0 | 58.6 | 59.9 | 1.0 |
|  | **Equatorial Guinea** | 5.6 | 2.7 | 2.1 | 25.5 | 25.1 | 1.0 |
|  | **Eritrea** | 3.8 | 0.0 | - | 12.9 | 12.7 | 1.0 |
|  | **Estonia** | 65.0 | 58.3 | 1.1 | 87.4 | 81.2 | 1.1 |
|  | **Eswatini** | 14.2 | 14.4 | 1.0 | 19.5 | 18.8 | 1.0 |
|  | **Ethiopia** | 4.9 | 4.6 | 1.1 | 20.6 | 19.8 | 1.0 |
|  | **Fiji** | 26.5 | 28.6 | 0.9 | 33.5 | 32.1 | 1.0 |
|  | **Finland** | 75.1 | 72.3 | 1.0 | 92.3 | 90.4 | 1.0 |
|  | **France** | 70.1 | 68.1 | 1.0 | 89.4 | 88.3 | 1.0 |
|  | **Gabon** | 13.3 | 12.0 | 1.1 | 26.1 | 25.0 | 1.0 |
|  | **Gambia** | 12.8 | 14.8 | 0.9 | 18.1 | 19.4 | 0.9 |
|  | **Georgia** | 50.7 | 48.7 | 1.0 | 53.8 | 52.3 | 1.0 |
|  | **Germany** | 73.7 | 72.5 | 1.0 | 87.8 | 87.3 | 1.0 |
|  | **Ghana** | 12.9 | 15.4 | 0.8 | 23.0 | 24.3 | 0.9 |
|  | **Greece** | 76.6 | 75.3 | 1.0 | 86.8 | 85.6 | 1.0 |
|  | **Greenland** | 43.6 | 42.7 | 1.0 | 60.2 | 59.7 | 1.0 |
|  | **Grenada** | 37.4 | 37.2 | 1.0 | 55.0 | 54.4 | 1.0 |
|  | **Guam** | 46.0 | 45.8 | 1.0 | 57.5 | 56.0 | 1.0 |
|  | **Guatemala** | 20.1 | 25.2 | 0.8 | 42.2 | 42.6 | 1.0 |
|  | **Guinea** | 6.7 | 9.9 | 0.7 | 12.6 | 14.0 | 0.9 |
|  | **Guinea-Bissau** | 6.1 | 5.6 | 1.1 | 12.4 | 13.1 | 0.9 |
|  | **Guyana** | 28.1 | 27.3 | 1.0 | 41.2 | 38.9 | 1.1 |
|  | **Haiti** | 8.7 | 11.1 | 0.8 | 21.3 | 21.1 | 1.0 |
|  | **Honduras** | 25.1 | 26.1 | 1.0 | 40.6 | 42.9 | 0.9 |
|  | **Hungary** | 52.0 | 49.5 | 1.1 | 73.6 | 71.9 | 1.0 |
|  | **Iceland** | 82.7 | 80.1 | 1.0 | 93.8 | 91.7 | 1.0 |
|  | **India** | 13.7 | 14.8 | 0.9 | 29.9 | 31.1 | 1.0 |
|  | **Indonesia** | 21.2 | 23.1 | 0.9 | 36.3 | 35.4 | 1.0 |
|  | **Iran (Islamic Republic of)** | 28.6 | 42.0 | 0.7 | 47.1 | 67.5 | 0.7 |
|  | **Iraq** | 19.0 | 31.0 | 0.6 | 37.1 | 55.9 | 0.7 |
|  | **Ireland** | 71.2 | 68.2 | 1.0 | 92.3 | 90.7 | 1.0 |
|  | **Israel** | 62.9 | 60.6 | 1.0 | 82.9 | 82.2 | 1.0 |
|  | **Italy** | 80.9 | 79.1 | 1.0 | 95.7 | 94.7 | 1.0 |
|  | **Jamaica** | 48.3 | 47.9 | 1.0 | 61.6 | 59.4 | 1.0 |
|  | **Japan** | 85.0 | 87.8 | 1.0 | 96.8 | 98.7 | 1.0 |
|  | **Jordan** | 23.4 | 36.0 | 0.6 | 45.9 | 65.5 | 0.7 |
|  | **Kazakhstan** | 40.8 | 37.7 | 1.1 | 57.2 | 53.5 | 1.1 |
|  | **Kenya** | 16.9 | 18.7 | 0.9 | 16.3 | 21.8 | 0.7 |
|  | **Kiribati** | 13.9 | 11.6 | 1.2 | 18.8 | 18.1 | 1.0 |
|  | **Kuwait** | 44.5 | 61.2 | 0.7 | 60.7 | 78.2 | 0.8 |
|  | **Kyrgyzstan** | 35.7 | 33.7 | 1.1 | 46.0 | 46.7 | 1.0 |
|  | **Lao People's Democratic Republic** | 8.7 | 10.1 | 0.9 | 26.5 | 26.1 | 1.0 |
|  | **Latvia** | 55.3 | 48.2 | 1.1 | 72.8 | 65.0 | 1.1 |
|  | **Lebanon** | 27.6 | 38.7 | 0.7 | 58.6 | 75.8 | 0.8 |
|  | **Lesotho** | 14.0 | 13.7 | 1.0 | 13.4 | 12.7 | 1.1 |
|  | **Liberia** | 7.6 | 9.2 | 0.8 | 16.5 | 18.9 | 0.9 |
|  | **Libya** | 25.7 | 39.4 | 0.7 | 38.1 | 57.5 | 0.7 |
|  | **Lithuania** | 56.4 | 57.8 | 1.0 | 68.8 | 68.9 | 1.0 |
|  | **Luxembourg** | 70.0 | 67.9 | 1.0 | 89.3 | 89.2 | 1.0 |
|  | **Madagascar** | 8.8 | 10.0 | 0.9 | 14.8 | 15.5 | 1.0 |
|  | **Malawi** | 9.8 | 11.8 | 0.8 | 18.0 | 17.6 | 1.0 |
|  | **Malaysia** | 30.4 | 31.3 | 1.0 | 56.9 | 57.3 | 1.0 |
|  | **Maldives** | 23.1 | 27.5 | 0.8 | 65.4 | 67.5 | 1.0 |
|  | **Mali** | 7.2 | 9.0 | 0.8 | 16.6 | 17.7 | 0.9 |
|  | **Malta** | 71.5 | 67.6 | 1.1 | 89.9 | 85.1 | 1.1 |
|  | **Marshall Islands** | 18.7 | 17.4 | 1.1 | 25.0 | 25.2 | 1.0 |
|  | **Mauritania** | 9.6 | 11.2 | 0.9 | 23.5 | 25.8 | 0.9 |
|  | **Mauritius** | 44.0 | 43.6 | 1.0 | 61.9 | 59.9 | 1.0 |
|  | **Mexico** | 38.1 | 39.9 | 1.0 | 61.9 | 62.9 | 1.0 |
|  | **Micronesia (Federated States of)** | 18.3 | 19.9 | 0.9 | 32.3 | 33.2 | 1.0 |
|  | **Monaco** | 80.9 | 79.2 | 1.0 | 89.4 | 87.5 | 1.0 |
|  | **Mongolia** | 22.1 | 22.3 | 1.0 | 37.4 | 35.5 | 1.1 |
|  | **Montenegro** | 62.6 | 60.0 | 1.0 | 71.6 | 69.8 | 1.0 |
|  | **Morocco** | 14.6 | 27.5 | 0.5 | 29.1 | 46.7 | 0.6 |
|  | **Mozambique** | 6.7 | 6.3 | 1.1 | 13.6 | 12.3 | 1.1 |
|  | **Myanmar** | 12.4 | 13.7 | 0.9 | 32.4 | 31.8 | 1.0 |
|  | **Namibia** | 13.7 | 14.0 | 1.0 | 29.2 | 29.1 | 1.0 |
|  | **Nauru** | 30.6 | 31.4 | 1.0 | 41.8 | 42.3 | 1.0 |
|  | **Nepal** | 6.3 | 7.7 | 0.8 | 24.6 | 24.6 | 1.0 |
|  | **Netherlands** | 78.6 | 75.9 | 1.0 | 91.6 | 90.6 | 1.0 |
|  | **New Zealand** | 82.0 | 79.5 | 1.0 | 93.9 | 93.7 | 1.0 |
|  | **Nicaragua** | 35.8 | 34.5 | 1.0 | 58.6 | 60.9 | 1.0 |
|  | **Niger** | 6.4 | 8.2 | 0.8 | 13.5 | 15.7 | 0.9 |
|  | **Nigeria** | 13.9 | 15.6 | 0.9 | 21.3 | 23.2 | 0.9 |
|  | **Niue** | 38.4 | 38.7 | 1.0 | 56.6 | 55.9 | 1.0 |
|  | **North Macedonia** | 47.2 | 45.5 | 1.0 | 65.2 | 63.5 | 1.0 |
|  | **Northern Mariana Islands** | 50.8 | 51.0 | 1.0 | 64.3 | 63.4 | 1.0 |
|  | **Norway** | 77.2 | 72.3 | 1.1 | 92.4 | 90.3 | 1.0 |
|  | **Oman** | 29.7 | 42.8 | 0.7 | 52.5 | 67.7 | 0.8 |
|  | **Pakistan** | 13.0 | 14.7 | 0.9 | 22.8 | 24.2 | 0.9 |
|  | **Palau** | 42.5 | 43.2 | 1.0 | 56.6 | 55.2 | 1.0 |
|  | **Palestine** | 24.9 | 36.5 | 0.7 | 37.3 | 54.2 | 0.7 |
|  | **Panama** | 48.8 | 49.0 | 1.0 | 67.7 | 68.0 | 1.0 |
|  | **Papua New Guinea** | 14.0 | 15.4 | 0.9 | 20.4 | 20.9 | 1.0 |
|  | **Paraguay** | 32.1 | 33.6 | 1.0 | 52.4 | 53.4 | 1.0 |
|  | **Peru** | 43.4 | 55.0 | 0.8 | 78.2 | 88.6 | 0.9 |
|  | **Philippines** | 28.5 | 31.5 | 0.9 | 39.9 | 40.6 | 1.0 |
|  | **Poland** | 32.4 | 31.4 | 1.0 | 56.7 | 55.3 | 1.0 |
|  | **Portugal** | 59.4 | 60.5 | 1.0 | 86.5 | 86.5 | 1.0 |
|  | **Puerto Rico** | 61.7 | 61.2 | 1.0 | 84.3 | 82.7 | 1.0 |
|  | **Qatar** | 27.3 | 39.6 | 0.7 | 54.2 | 69.6 | 0.8 |
|  | **Republic of Korea** | 59.0 | 60.0 | 1.0 | 91.6 | 93.6 | 1.0 |
|  | **Republic of Moldova** | 47.6 | 44.7 | 1.1 | 65.4 | 62.0 | 1.1 |
|  | **Romania** | 49.6 | 46.4 | 1.1 | 73.0 | 67.5 | 1.1 |
|  | **Russian Federation** | 56.9 | 42.9 | 1.3 | 73.3 | 61.3 | 1.2 |
|  | **Rwanda** | 4.1 | 3.6 | 1.2 | 21.0 | 19.9 | 1.1 |
|  | **Saint Kitts and Nevis** | 47.4 | 45.8 | 1.0 | 65.1 | 63.5 | 1.0 |
|  | **Saint Lucia** | 39.0 | 37.3 | 1.0 | 59.0 | 56.6 | 1.0 |
|  | **Saint Vincent and the Grenadines** | 39.9 | 39.7 | 1.0 | 50.2 | 48.3 | 1.0 |
|  | **Samoa** | 31.9 | 31.0 | 1.0 | 43.6 | 42.8 | 1.0 |
|  | **San Marino** | 78.3 | 76.3 | 1.0 | 88.5 | 87.7 | 1.0 |
|  | **Sao Tome and Principe** | 14.2 | 12.7 | 1.1 | 25.7 | 25.0 | 1.0 |
|  | **Saudi Arabia** | 6.1 | 34.0 | 0.2 | 47.1 | 71.5 | 0.7 |
|  | **Senegal** | 10.0 | 10.7 | 0.9 | 17.1 | 18.6 | 0.9 |
|  | **Serbia** | 38.2 | 50.7 | 0.8 | 59.2 | 73.5 | 0.8 |
|  | **Seychelles** | 33.5 | 32.6 | 1.0 | 53.4 | 53.4 | 1.0 |
|  | **Sierra Leone** | 9.0 | 9.8 | 0.9 | 14.5 | 17.2 | 0.8 |
|  | **Singapore** | 70.1 | 71.9 | 1.0 | 93.3 | 95.0 | 1.0 |
|  | **Slovakia** | 64.2 | 59.8 | 1.1 | 83.1 | 78.9 | 1.1 |
|  | **Slovenia** | 61.6 | 59.6 | 1.0 | 82.4 | 80.3 | 1.0 |
|  | **Solomon Islands** | 20.4 | 21.8 | 0.9 | 30.1 | 31.3 | 1.0 |
|  | **Somalia** | 5.3 | 5.6 | 0.9 | 7.3 | 8.9 | 0.8 |
|  | **South Africa** | 22.7 | 25.1 | 0.9 | 33.6 | 33.9 | 1.0 |
|  | **South Sudan** | 6.7 | 6.5 | 1.0 | 10.5 | 10.6 | 1.0 |
|  | **Spain** | 77.6 | 75.1 | 1.0 | 93.9 | 92.0 | 1.0 |
|  | **Sri Lanka** | 36.5 | 36.6 | 1.0 | 65.2 | 64.8 | 1.0 |
|  | **Sudan** | 10.7 | 20.8 | 0.5 | 22.7 | 38.1 | 0.6 |
|  | **Suriname** | 32.4 | 31.2 | 1.0 | 46.4 | 44.2 | 1.0 |
|  | **Sweden** | 78.1 | 75.5 | 1.0 | 88.7 | 88.5 | 1.0 |
|  | **Switzerland** | 89.5 | 80.0 | 1.1 | 99.1 | 92.9 | 1.1 |
|  | **Syrian Arab Republic** | 25.9 | 36.1 | 0.7 | 41.4 | 60.2 | 0.7 |
|  | **Taiwan (Province of China)** | 64.7 | 63.6 | 1.0 | 86.5 | 86.6 | 1.0 |
|  | **Tajikistan** | 31.4 | 31.5 | 1.0 | 32.5 | 32.5 | 1.0 |
|  | **Thailand** | 34.3 | 34.7 | 1.0 | 66.3 | 66.1 | 1.0 |
|  | **Timor-Leste** | 12.6 | 16.2 | 0.8 | 29.5 | 28.8 | 1.0 |
|  | **Togo** | 12.1 | 14.2 | 0.9 | 18.4 | 19.9 | 0.9 |
|  | **Tokelau** | 25.0 | 26.7 | 0.9 | 45.2 | 44.8 | 1.0 |
|  | **Tonga** | 29.3 | 32.2 | 0.9 | 39.2 | 40.4 | 1.0 |
|  | **Trinidad and Tobago** | 39.7 | 37.9 | 1.0 | 59.7 | 55.5 | 1.1 |
|  | **Tunisia** | 28.0 | 40.5 | 0.7 | 49.3 | 67.2 | 0.7 |
|  | **Turkey** | 18.5 | 30.7 | 0.6 | 49.2 | 69.9 | 0.7 |
|  | **Turkmenistan** | 33.7 | 33.4 | 1.0 | 45.7 | 44.5 | 1.0 |
|  | **Tuvalu** | 20.2 | 21.6 | 0.9 | 34.5 | 35.2 | 1.0 |
|  | **Uganda** | 10.6 | 10.7 | 1.0 | 19.8 | 19.0 | 1.0 |
|  | **Ukraine** | 54.2 | 45.1 | 1.2 | 67.8 | 57.0 | 1.2 |
|  | **United Arab Emirates** | 23.5 | 29.6 | 0.8 | 37.7 | 47.2 | 0.8 |
|  | **United Kingdom** | 72.7 | 70.8 | 1.0 | 89.0 | 88.8 | 1.0 |
|  | **United Republic of Tanzania** | 12.0 | 11.9 | 1.0 | 20.8 | 20.5 | 1.0 |
|  | **United States of America** | 87.6 | 86.6 | 1.0 | 94.4 | 94.2 | 1.0 |
|  | **United States Virgin Islands** | 48.4 | 46.8 | 1.0 | 64.1 | 61.9 | 1.0 |
|  | **Uruguay** | 45.7 | 46.1 | 1.0 | 62.2 | 61.7 | 1.0 |
|  | **Uzbekistan** | 41.7 | 41.1 | 1.0 | 43.4 | 44.6 | 1.0 |
|  | **Vanuatu** | 18.5 | 18.8 | 1.0 | 22.8 | 23.1 | 1.0 |
|  | **Venezuela (Bolivarian Republic of)** | 40.6 | 41.2 | 1.0 | 63.4 | 63.8 | 1.0 |
|  | **Viet Nam** | 28.5 | 26.9 | 1.1 | 58.7 | 56.0 | 1.0 |
|  | **Yemen** | 10.9 | 20.8 | 0.5 | 18.6 | 33.5 | 0.6 |
|  | **Zambia** | 7.0 | 7.4 | 0.9 | 21.0 | 19.8 | 1.1 |
|  | **Zimbabwe** | 21.8 | 21.6 | 1.0 | 18.4 | 17.0 | 1.1 |

Abbreviations: QCI=Quality of care index; WHO=World health organization; GDR=gender disparity ratio; SDI=socio-demographic index.
