# Supplementary figures and images for: The global, regional, and national burden and quality of care index (QCI) of colorectal cancer; a global burden of disease systematic analysis 1990–2019
Source: PLoS One. 2022 Apr 21;17(4):e0263403. doi: 10.1371/journal.pone.0263403 (PMC9022854; doi:10.1371/journal.pone.0263403)

1990  
Female

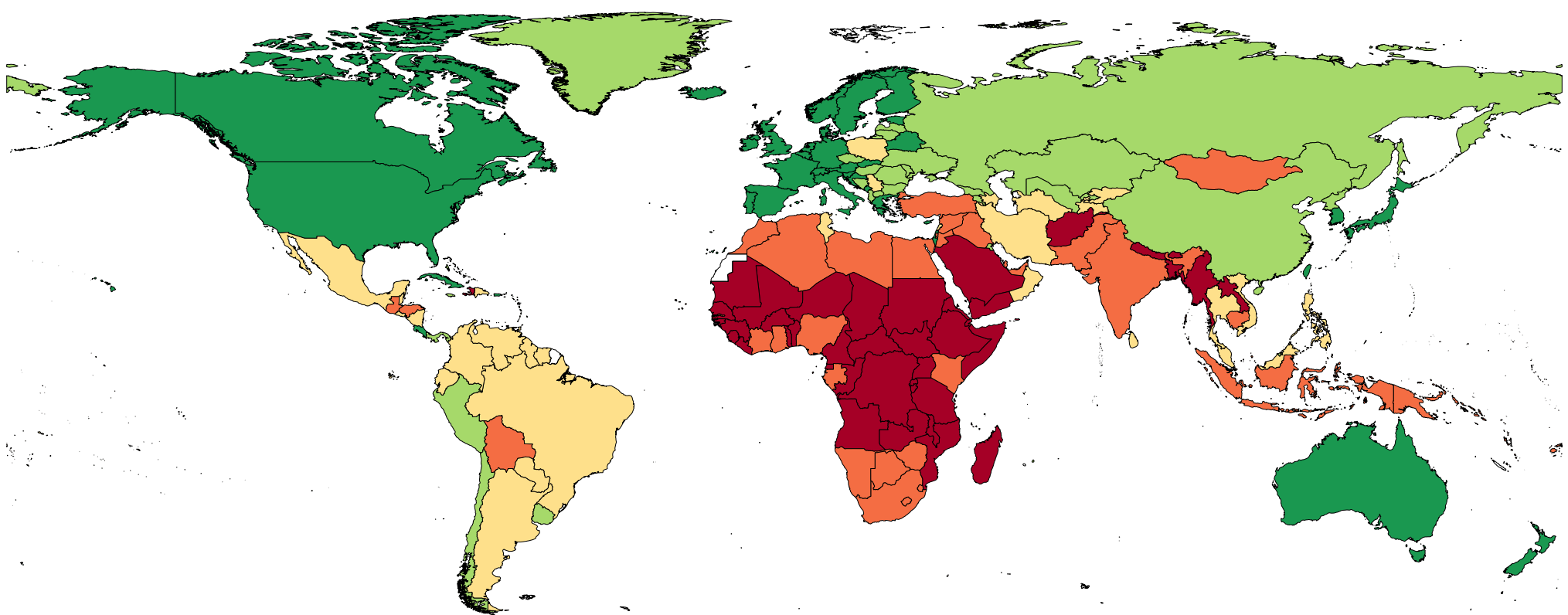

Male

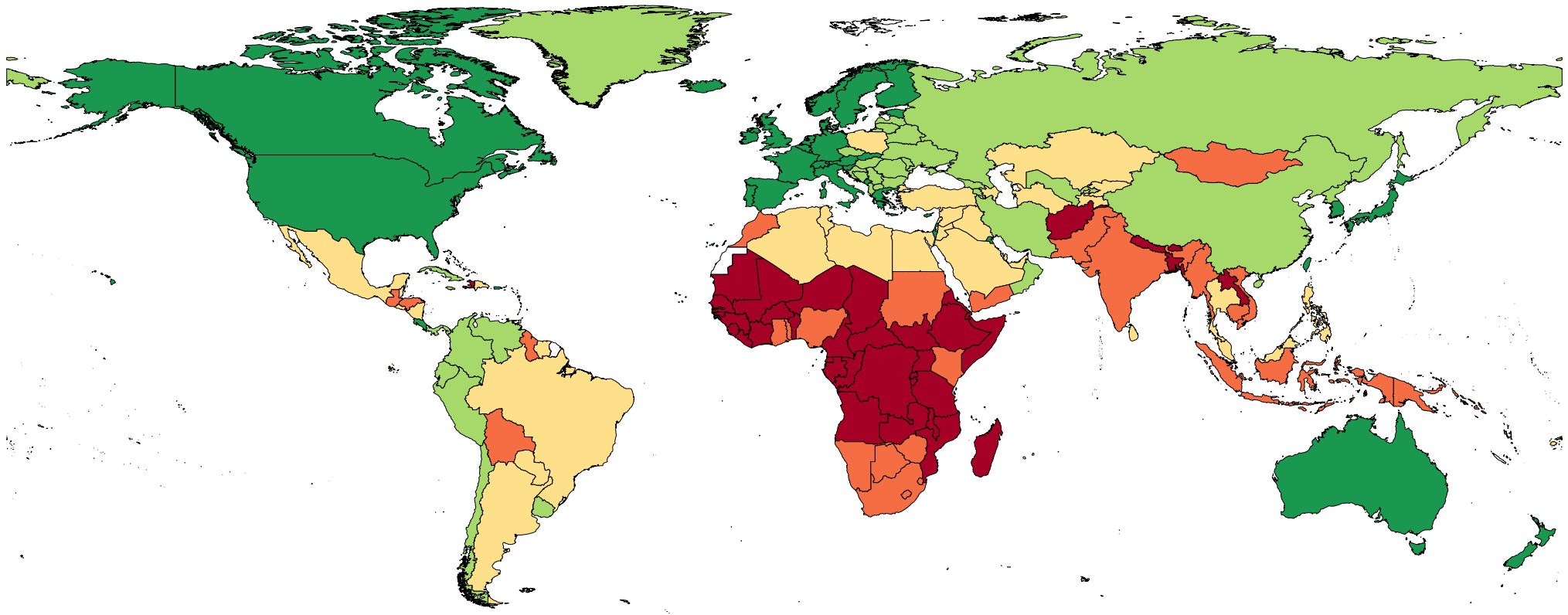

Age-standardized QCI (%)

- < 12.5
- [12.5 to 27.6)
- [27.6 to 40.7)
- [40.7 to 58.0)
- ≥ 58.0

Supplement: S1 Fig — (PDF) [file pone.0263403.s006.pdf]

2019

Female

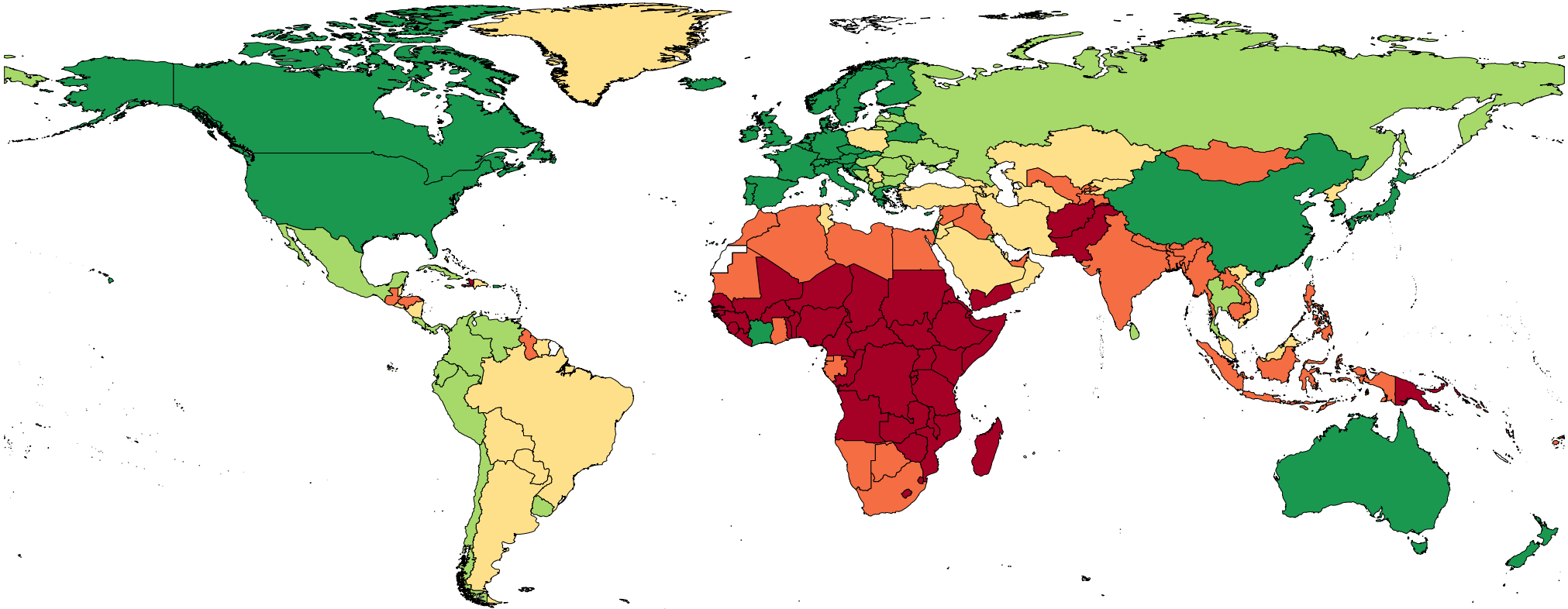

Male

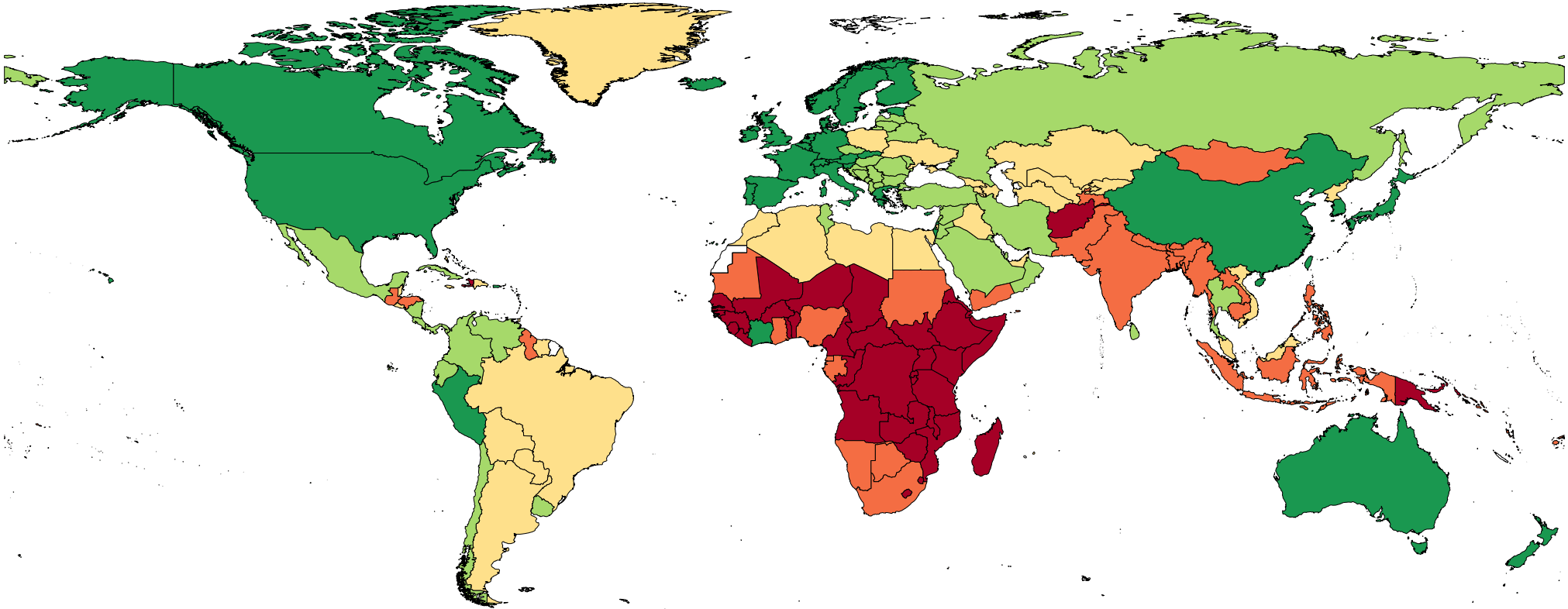

Age-standardized QCI (%)

- < 22.8
- [22.8 to 44.1)
- [44.1 to 60.2)
- [60.2 to 78.4)
- ≥ 78.4

Supplement: S2 Fig — (PDF) [file pone.0263403.s007.pdf]
