# Supplementary material for: The global, regional, and national burden and quality of care index (QCI) of colorectal cancer; a global burden of disease systematic analysis 1990–2019
Source: PLoS One. 2022 Apr 21;17(4):e0263403. doi: 10.1371/journal.pone.0263403 (PMC9022854; doi:10.1371/journal.pone.0263403)
Supplement: S1 Appendix — (DOCX) [file pone.0263403.s008.docx]

**S1 Appendix:**

**Details of mathematical calculation of quality of care index.**

**Quality of care index (QCI)**

The mixed effect formula for validation of QCI was:

$$Y=X_{f}\beta+Z_{r}U+\varepsilon$$

$X_{f}$=is the explanatory parameter with fixed effect of $\beta$

$Z_{r}$=is the explanatory parameter with random effect of $U$

The QCI formula was considered for each sex (both, male, female), age groups (5 to 80+ years, all ages, and age-standardized), year (1990 to 2019, annually), and location (Global, 6 WHO regions, 21 GBD regions, 5 SDI quintiles, and 204 countries). Further details on the analysis are provided below as figures with descriptions added as legends:


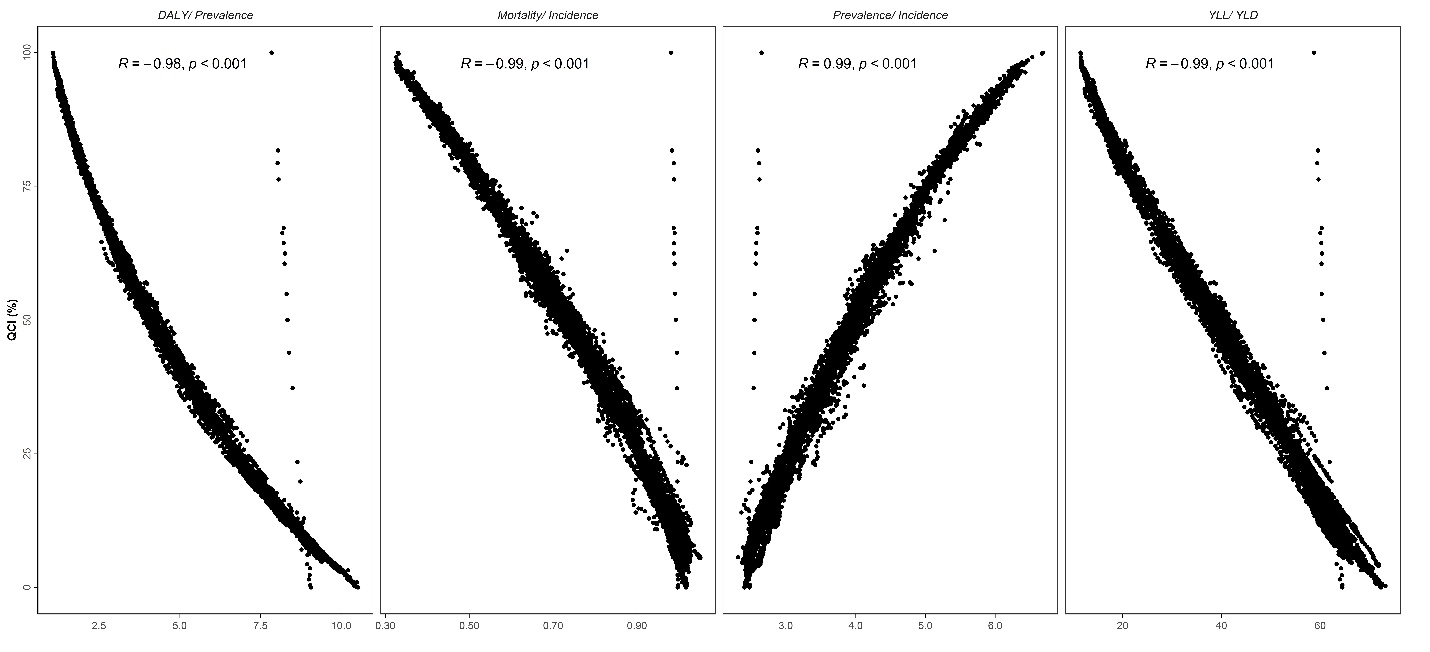


Figure 1. The correlation coefficients for all of the four entry variables with QCI is greater than 0.98. Just the Prevalence-to-incidence ratio had an inverse relation as it was anticipated. It should be reminded that in case of constant incidence, regions with higher prevalence rate have provided better care to their population.

**
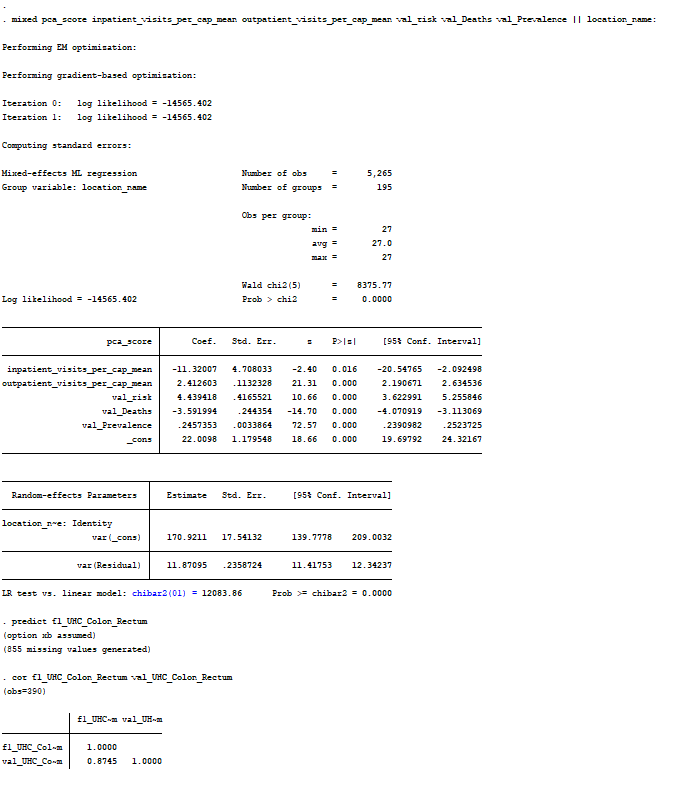
**

Figure 2. Based on the fitted mixed-effect model (above), the estimated variance of the constant per country - the individual level random effect is non-zero (and not close to zero), that is evidence that the random effect is beneficial.

The χ2 test is a formal test of the inclusion of the random effects versus without the random effects. We reject the null that the models are equivalent, so it is appropriate to include the random effects (P-value<0.001).


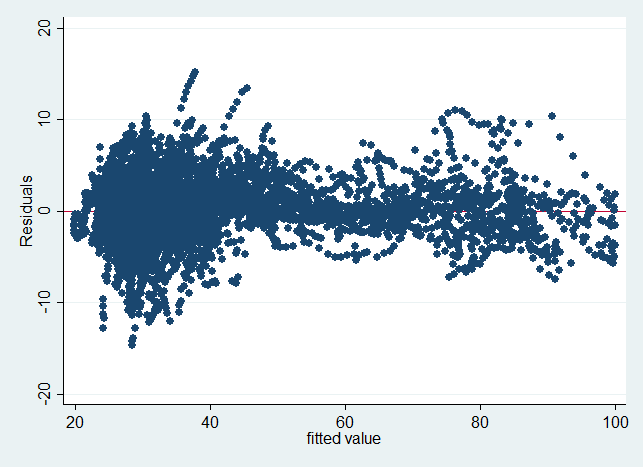


Figure 3. The scatter plot between the fitted values and their residuals is presented. We’re looking for either a blatant deviation from a mean of 0, or an increasing/decreasing variability on the y-axis over the fitted value.


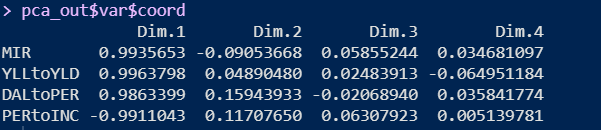


Figure 4. The slopes of each of four entry variables in each of dimensions.


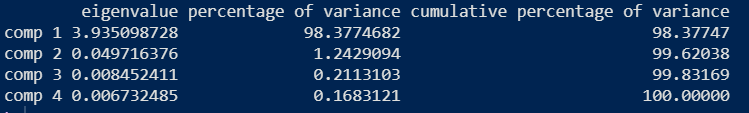


Figure 5. The eigenvalue of the first component was close to 4 (as the largest possibility in a 4-dimension PCA) and has grabbed 98.4% of variability and information of total data points in age-standardized both-sex population.


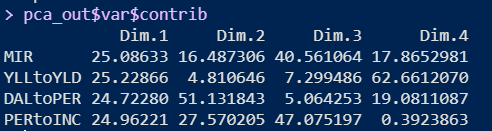


Figure 6. Percent of contribution of each of four secondary variables (as entries) with different components [=dimensions] of PCA on age-standardized both-sex subpopulation of colorectal cancer from 1990 to 2019.


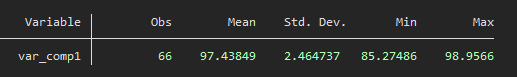


Figure 7. The characteristics of the first component of PCA on age-standardized both sexes. The mean variance is 97.4, ranging from 85.27 to 98.96.
